# Transdermal bicarbonate buffer therapy increases intratumoral pH and elicits antitumor responses in bladder cancer

**DOI:** 10.3389/fimmu.2026.1706250

**Published:** 2026-03-13

**Authors:** Oluwaseyi Oluwatola, Sarah Bazargan, Pietro Irrera, Darwin Chang, Ashley Thomas, Jamie Blauvelt, Matthew Beatty, MacLean Hall, Christopher J. Whelan, Veronica Estrella, Katarzyna A. Rejniak, Michael Poch, Nathan Fitzsimmons, Ryan Beal, Arig Ibrahim-Hashim, Shari Pilon-Thomas

**Affiliations:** 1 Department of Immunology, H. Lee Moffitt Cancer Center & Research Institute, Tampa, FL, United States; 2 Department of Molecular Medicine, University of South Florida, Tampa, FL, United States; 3 College of Arts and Sciences, University of South Florida, Tampa, FL, United States; 4 Department of Metabolism and Physiology, H. Lee Moffitt Cancer Center & Research Institute, Tampa, FL, United States; 5 Department of Biological Sciences, University of Illinois, Chicago, IL, United States; 6 Department of Integrated Mathematical Oncology, H. Lee Moffitt Cancer Center & Research Institute, Tampa, FL, United States; 7 Genitourinary Oncology Program, H. Lee Moffitt Cancer Center & Research Institute, Tampa, FL, United States; 8 Dyve Biosciences, Inc., Camarillo, CA, United States; 9 Faculty of Education and Arts, Sohar University, Sohar, Oman

**Keywords:** acidity, bladder cancer, DYV800, pH, T cell, transdermal therapy, tumor microenvironment

## Abstract

**Introduction:**

Tumor acidosis is a hallmark of cancer that leads to abrogation of T cell function and cancer progression. Oral sodium bicarbonate therapy for alkalization of the extracellular tumor pH has shown moderate positive effects in tumor models. However, its applicability in the clinic is very limited due to the unreasonably high dosage required and gastrointestinal disturbances that arise. In this study, we assessed the functional effects of acidity on T cells.

**Methods:**

We show that acidity alters T cell proliferation, migration and effector functions as well as transcriptional programming using in vitro culture techniques and RNA sequencing. We then tested the potency of a proprietary transdermal formulation, DYV800, containing sodium bicarbonate to increase the extracellular tumor pH (pHe) and augment anti-tumor immune responses in a murine model of bladder cancer. The tumor pH was assessed using Chemical Exchange Saturation Transfer Magnetic Resonance Imaging (CEST-MRI) and antitumor immune responses via flow cytometry.

**Results:**

We found that transdermal DYV800 significantly reduced tumor burden and improved antigen-specific CD8^+^ T cell responses. Chemical Exchange Saturation Transfer Magnetic Resonance Imaging (CEST-MRI) of treated tumors showed an increase in intra-tumoral pH of bladder tumors, and this therapy also alkalizes the urine.

**Discussion/Conclusion:**

The transdermal delivery of DYV800 led to durable anti-tumor immune responses and is more clinically applicable to combat acidity in bladder cancer than oral bicarbonate. Targeting acidosis in the bladder tumor microenvironment has the potential to enhance T cell responses and improve anti-tumor immunity.

## Introduction

The acidic microenvironment represents a hallmark of solid tumors, with profound implications for cancer progression, therapeutic resistance, and immune evasion (1–3). Tumor acidification is largely driven by the metabolic reprogramming of cancer cells, which rely heavily on glycolysis for energy production even in oxygen-rich conditions—a phenomenon known as the Warburg effect (4–6). The resulting acidosis, with pH (6.2–6.8) compared to the physiological pH of 7.4, creates a hostile environment that favors tumor progression (7, 8).

Bladder cancer is characterized by high recurrence rates and provides a unique model for studying the effects of acidosis on tumor biology and therapeutic responses (9). Unlike most solid tumors, bladder tumors experience dual acidification: intrinsic acidification from aberrant cancer metabolism and continuous exposure to acidic urine (pH 5.5–7.0) (10). Studies demonstrate that consistently acidic urine (<pH 6.0) is associated with increased bladder cancer risk (10) and renal failure (11, 12). This distinctive acidic microenvironment may contribute to the aggressive nature and treatment resistance of bladder cancers, particularly in muscle-invasive and advanced disease.

Given the critical role of acidosis in cancer progression and immunosuppression, therapeutic strategies targeting acidity have emerged as promising adjuncts to conventional treatments. Systemic buffering using oral sodium bicarbonate has demonstrated efficacy in preclinical models by neutralizing extracellular acidity and improving antitumor responses. One of the first studies conducted shows that sodium bicarbonate in water effectively neutralizes tumor acidity without impacting systemic pH (13, 14). Other studies show effectiveness in melanoma (15), prostate cancer prevention in TRAMP mice (13, 16), and enhanced immunotherapy responses when combined with checkpoint blockade (17). However, early-phase clinical trials testing oral sodium bicarbonate tolerability in pancreatic cancer patients and as adjuvant therapy for tumor-related pain failed due to dosing issues, poor compliance from unpalatable taste, and gastrointestinal disturbances (18). Additionally, interspecies allometric scaling indicates that the required human dose (16.3 g/day, equivalent to 4.2 mL/day in mice at 2.8 g/kg/day) is not feasible (14). These limitations necessitate testing alternative, clinically applicable delivery routes to modulate systemic pH.

In this paper, we first characterize the effects of acidity on CD8^+^ T cells through *in vitro* experiments and RNA sequencing. Second, we investigate the ability of DYV800, a novel transdermal bicarbonate therapy that neutralizes pH systematically, improves antitumor T-cell responses, and prevents tumor progression. This study provides compelling evidence for a pH-modulating strategy in bladder cancer and offers insights into broader applications of transdermal buffer therapy in cancer treatment.

## Materials and methods

### Mice and cell lines

Female C57BL/6 mice (8 weeks old; RRID: IMSR_JAX:000664) were obtained from the Charles River Laboratory. OT-I (C57BL/6-Tg(TcraTcrb)1100Mjb/J; RRID: IMSR_JAX:003831) and pmel (B6.Cg-Thy1a/Cy Tg(TcraTcrb)8Rest/J) transgenic mice were purchased from The Jackson Laboratory. All mice used in the experiment were housed on the same rack within the Comparative Medicine Facility at Moffitt Cancer Center and handled consistently by the same staff, and measurements were completed as scheduled. Mice were humanely euthanized with CO^2^ inhalation for tissue collection at endpoint tumor sizes (area of 200 m^2^ or volume of 250 mm^3^ for subcutaneous and orthotopic tumors, respectively). The MB49 murine bladder cancer cell line was provided by Dr. Jeffery Schlom (National Cancer Institute). The MB490VA variant was established as described previously (19). All cell lines were maintained in RPMI Complete Media (CM) supplemented with 10% FBS and passaged less than 10 times to maintain genomic stability and prevent contamination. All cell lines tested negative for mycoplasma.

### *In vitro* experiments

#### T-cell isolation from splenocytes and stimulation

Splenocytes from OT-I mice were prepared by mechanically dissociating spleens through 100-μm filters. Cell suspensions were washed with HBSS (Corning, Corning, New York, ref# 21-021-CM) and centrifuged at 500*g* for 5 min at 4 °C. Red blood cells were eliminated by incubating cell pellets in 5–10 mL of RBC lysis buffer (BioLegend, San Diego, California, USA, 420301) for 5 min. After centrifugation, splenocytes were resuspended in CM. CD8^+^ T or total T cells were isolated from splenocytes using the EasySep Mouse CD8^+^ T/T Cell Isolation Kit (StemCell, Cambridge, Massachusetts, USA, cat# 19853/cat# 19851A, respectively) according to the manufacturer’s instructions. OT-I T cells were activated using OVA (257-264) peptide fragment (SIINFEKL) (AnaSpec, Fremont, California, USA, cat# AS-60193-5) at 5 μg/mL. Pmel T cells were stimulated with human gp100 (25-33) peptide (AnaSpec, AS-62589) at 5 μg/mL and supplemented with recombinant human IL-2 (Akron, Boca Raton, Florida, USA, cat# AR1002-0022) at 10 IU/mL. T cells from C57BL/6 were stimulated with cell activation cocktail (BioLegend, #423303) for 6 h.

#### Preparation of pH 7.4 and 6.6 media

CM was prewarmed to 37°C and divided into two. pH 6.6 medium was supplemented with 1 M of PIPES (Thermo Scientific, Waltham, Massachusetts, USA, cat# J62195.AK) to 25 mM final concentration; pH 7.4 medium was supplemented with 1 M of HEPES (Corning, ref# 25-060-CI) to 25 mM. pH values were adjusted using 1 N of NaOH (pH 7.4) or 1 N of HCl (pH 6.6) and measured using an Accumet AB150 pH benchtop meter (Thermo Scientific). Both media were sterilized through 0.22-μm filters (Corning) and then placed overnight in 5% CO^2^ incubators for CO^2^ equilibration. pH values were verified and readjusted, if necessary, the following day, and media were refiltered. Fresh media were prepared for each experiment.

#### Proliferation assays

CellTrace Violet (Invitrogen, Carlsbad, California, USA, ref# C34557) was reconstituted with 20 μL of DMSO, and then 10 μL was diluted into 5 mL of prewarmed PBS. Cells were pelleted by centrifugation (500*g*, 4°C, 5 min) and combined with ≥1 mL of CTV solution. Cell suspensions were incubated at 37°C in the dark for 20 min and then washed with 5–10 mL of CM. Labeled cells were divided, resuspended in pH-adjusted media, stimulated with corresponding peptides, and plated. Unstimulated CTV-labeled cells and unlabeled cells served as controls and were stained for viability using Live/Dead reagent and for CD8^+^ and CD4^+^ T-cell markers. At 72 h post-plating, all cells were collected and processed for staining. For the thymidine incorporation assay, T cells were stimulated under respective pH conditions in 96-well plates and supplemented with 1 μCi of ³H-thymidine 12–18 h before harvest. Cells were harvested onto filter mats (PerkinElmer, Shelton, Connecticut, USA), dried at 60°C for up to 1 h, heat-sealed in plastic sleeves, and saturated with BetaPlate scintillation fluid (PerkinElmer). Radioactivity was quantified using a MicroBeta Microplate Counter (PerkinElmer), with the results expressed as counts per minute (CPM).

#### Tumor cell coculture

Isolated CD8^+^ OT-I T cells and MB49/MB49OVA tumor cells were cocultured at a 10:1 ratio for 24 h under pH 7.4 or 6.6 conditions. To enhance MHC class I expression, cell lines were pretreated with 50 ng/mL of murine IFN-γ (Peprotech, Waltham, Massachusetts, USA, cat# 315-05-100UG) for 48 h. After 18 h, T cells were treated with brefeldin A (BioLegend, cat# 420601), harvested, and stained for intracellular IFN-γ by flow cytometry.

#### Cytometric bead array

T cells were activated and cultured under specified pH conditions as previously described. Culture supernatants were collected at designated timepoints and stored at −20°C for cytokine analysis. The LEGENDplex Mouse B cell Panel (13-plex) with filter plate (BioLegend, cat# 740818) was used according to the manufacturer’s instructions. Data were acquired on a BD FACSCelesta flow cytometer (BD Biosciences, Franklin Lakes, New Jersey (NJ), USA) and analyzed using the LEGENDplex Data Analysis Software Suite (Qognit, BioLegend).

#### Migration assay

Isolated T cells (from C57BL/6 mice) were resuspended at 1 × 10^7^ cells/mL in pH 7.4 or pH 6.6 media at 37°C before being loaded into Transwell plates (Costar, Corning, New York (NY), USA, ref# 3421). CXCL10 (Peprotech, cat# 250-16) was prepared in serial dilutions (1,000–62.5 ng/mL) in both pH media. CXCL10-containing and control media were loaded into the bottom chambers and incubated for 30 min for chemokine equilibration. Then, 100 μL of cell suspensions were pipetted into Transwell inserts and transferred to wells. For loading controls, 20 μL of cell suspension was placed directly into 3–4 wells (without inserts) per pH condition. Plates were incubated at 37°C for 2 h. After incubation, the media containing migrated cells were washed and resuspended in flow buffer. Each sample was supplemented with 1 × 10^4^ CountBright Plus Absolute Counting Beads (Invitrogen, ref# C36995) and analyzed by flow cytometry. Migrating cells were calculated as follows: [(cell counts)/(bead counts)] × 10^4^.

#### RNA sequencing and analysis

Isolated CD8^+^ pmel T cells were plated in pH 7.4 or pH 6.6 media. After a 48-h incubation, RNA was extracted using the RNeasy Mini Kit (Qiagen, Valencia, California (CA), USA, cat# 74104). RNA quantity and purity were assessed using a NanoDrop spectrophotometer (Thermo Fisher, Waltham, Massachusetts, USA), and five optimal samples per condition were selected. RNA concentration was quantified using a Qubit Fluorometer (Thermo Fisher Scientific), and integrity was assessed using Agilent TapeStation 4200 (Agilent Technologies, Santa Clara, California (CA), USA; RRID: SCR_019547). RNA-seq libraries were prepared using NuGEN Universal Mouse RNA-Seq Library Preparation Kit (Tecan Genomics, San Jose, California (CA), USA) with 100 ng of input RNA. Library quality was confirmed via TapeStation and quantified using the KAPA Library Quantification Kit (Roche, Nutley, New Jersey (NJ), USA). Libraries were sequenced on the Illumina NovaSeq 6000 platform using the SP 200-cycle reagent kit, generating ~100 million 100-base paired-end reads per sample. Adapter sequences were removed using BBMerge (v37.02) and Cutadapt (v1.8.1; RRID: SCR_011841). Reads were aligned to the mouse genome (mm10) using the STAR aligner (v2.7.7a), and gene expression was quantified using RSEM (v1.3.0) with GENCODE annotation. Differential expression analysis was performed using DESeq2 (RRID: SCR_000154) on genes with base mean >100. Results were analyzed using Qiagen’s Ingenuity Pathway Analysis (IPA; RRID: SCR_008653) for genes with padj <0.05 and |log2fold change| >1. Downregulated genes (padj < 0.05, log2fold change < -1) were analyzed using ShinyGO v0.77 (RRID: SCR_019213) against murine TF.Target.RegNetwork database. Gene set enrichment analysis (GSEA v4.2.3, Broad Institute, Cambridge, Massachusetts, USA) was performed on complete datasets using MSigDB Hallmark gene sets.

#### Cell cycle analysis

OT-I T cells were stimulated as previously described and harvested at 24 h post-stimulation. Cell cycle analysis was performed using the Cell Cycle Analysis Kit (Abcam, Branford, Connecticut (CT), USA, cat# AB287852). Cells were treated with 1× ice-cold cell cycle buffer, centrifuged, and fixed dropwise with ice-cold 70% ethanol while gently vortexing. Fixed cells were stored overnight at 4 °C and then transferred to −20°C. On staining day, samples were thawed, centrifuged, washed twice with ice-cold 1× cell cycle buffer, and stained in the dark at room temperature for 45 min. Samples were immediately acquired on a BD FACSCanto flow cytometer (BD Biosciences) and analyzed using FlowJo (RRID: SCR_008520) and ModFit LT (Verity Software House).

### *In vivo* experiments

#### Tumor inoculation and treatment with DYV800 cream

For subcutaneous tumors, mice were injected subcutaneously into the right flank with 1 × 10^5^ MB49OVA cells and randomized into control and DYV800 treatment groups, and treatment was initiated 24 h or 7 days post-tumor inoculation (5–10 mice/group). Tumor size was measured with calipers and reported as mm^2^. Orthotopic tumors were established as previously described (19). Orthotopic tumor establishment and growth were monitored using the Vevo F2 imaging system (FUJIFILM VisualSonics). Image stacks were acquired and analyzed with Vevo LAB software to calculate tumor volumes and reported as mm^3^. Mice with confirmed orthotopic tumors were randomized for the experiments. Mice without observable tumors were omitted from the experiment prior to randomization and the start of treatment. Tumor-bearing mice were randomly assigned to the treatment or control groups. DYV800 cream was provided by Dyve Biosciences Inc., Camarillo, CA 93012, USA After tumor implantation, 100 μL of cream was applied twice daily on the flank (subcutaneous tumors) or abdomen (orthotopic tumors) until endpoint. At the endpoint, the mice were euthanized and the tumors were collected.

#### Flow cytometry staining

Extracted tumors were minced and enzymatically digested in HBSS buffer containing 1 mg/mL of collagenase, 0.1 mg/mL of DNase I, and 2.5 U/mL of hyaluronidase (Sigma-Aldrich, St. Louis, Missouri (MO), USA). Tissue suspensions were processed using gentleMACS Dissociator (Miltenyi Biotec, Gaithersburg, Maryland (MD), USA) and incubated at 37°C for 45 min. Following incubation, samples were filtered (100 μm strainer), centrifuged, treated with red blood cell lysis buffer, and filtered through a 70-μm cell strainer. Viability staining of cells was performed using Zombie NIR Fixable Viability Dye (BioLegend, cat# 423106) in PBS for 30 min at room temperature. Cells were washed with flow buffer and blocked with anti-mouse CD16/CD32 (Tonbo Biosciences, San Diego, California (CA), ref# 70-0161-M001) for 10 min at 4°C. Surface staining was performed by incubating cells for 20 min at 4°C with mouse-reactive antibodies. Then, cells were fixed in 2%–4% paraformaldehyde for 15 min at 4 °C. For intracellular staining, cells were permeabilized using 1× BD Perm/Wash Buffer (BD, cat# 554723) for 15 min at 4 °C and stained for 30 min at 4°C. Cells were washed twice with Perm/Wash buffer and resuspended in flow buffer. The staining was done in the dark. See **Table 1** for the flow antibodies used. Samples were acquired on BD FACSCelesta (BD Biosciences) and analyzed using FlowJo version 10.9.0 (RRID: SCR_008520).

**Table 1:** List of Antibodies used.

Fluorochrome	Antigen	Catalog number	Supplier
BV786	CD8a	100750	BioLegend
PerCP-Cy5.5	CD4	116012	BioLegend
BV650	CD69	104541	BioLegend
BV510	CD25	102042	BD Pharmingen
PE	CXCR3	126506	BioLegend
PE-Cy7	CD103	121436	BioLegend
PE	FasL	106605	BioLegend
PE	H-2Kb/SIINFEKL	141604	BioLegend
PE	I-A^b^ PE	553552	BD Pharmingen
Pacific Blue	CD4	558107	BD Pharmingen
BV605	CD45	103140	BioLegend
BV650	4-1BB	740499	BDOptiBuild
BV421	PD-1	135221	BioLegend
PE-Cy7	NK1.1	108714	BioLegend
PE	TIM-3	134004	BioLegend
PE	IFN-γ	554412	BD Pharmingen
FITC	Granzyme B	396404	BioLegend
BV421	TNF-α	563387	BDHorizon

#### Urine pH measurements

After tumor implantation, free catch urine was collected by applying gentle pressure to the bladder area, and pH was assessed using 10-parameter urine test strips (Avantor, Radnor, Pennsylvania (PA), USA ,URS-10PR-7777). Baseline urine pH was recorded before and after DYV800 treatment, then at the indicated timepoints.

#### CEST imaging

Mice were anesthetized and catheters were inserted for contrast delivery as previously described (20). A 7-T horizontal magnet (Agilent ASR 310; Santa Clara, CA) (Bruker Biospin, Inc. BioSpect AV3HD, Billerica, MA) with a 1H 30 mm M2M coil was used for imaging. CEST images were collected once pre-contrast and four times post-contrast using an IV + infusion protocol (200 μL of bolus followed by 400 μL/h infusion, total volume 320–330 μL) over ≈18 min post-contrast acquisition time to ensure constant tumor agent concentration. CEST images were analyzed using MATLAB (RRID: SCR_001622) with a multipool Lorentzian fitting script to establish iopamidol contribution at 4.2 and 5.5 ppm (21).

### Statistical analysis

All graphs were generated using GraphPad Prism version 9.2.0 (GraphPad Software Inc., San Diego, California (CA), USA; RRID: SCR_002798), unless otherwise stated. Unless specified otherwise, error bars represent the standard deviation of technical replicates. Analysis between two groups was done by a two-tailed independent *t*-test or two-way ANOVA (as indicated in the figure legends). Statistical significance was set as *p* < 0.05.

## Results

### Acidity inhibits T-cell function

**Figure 1:**
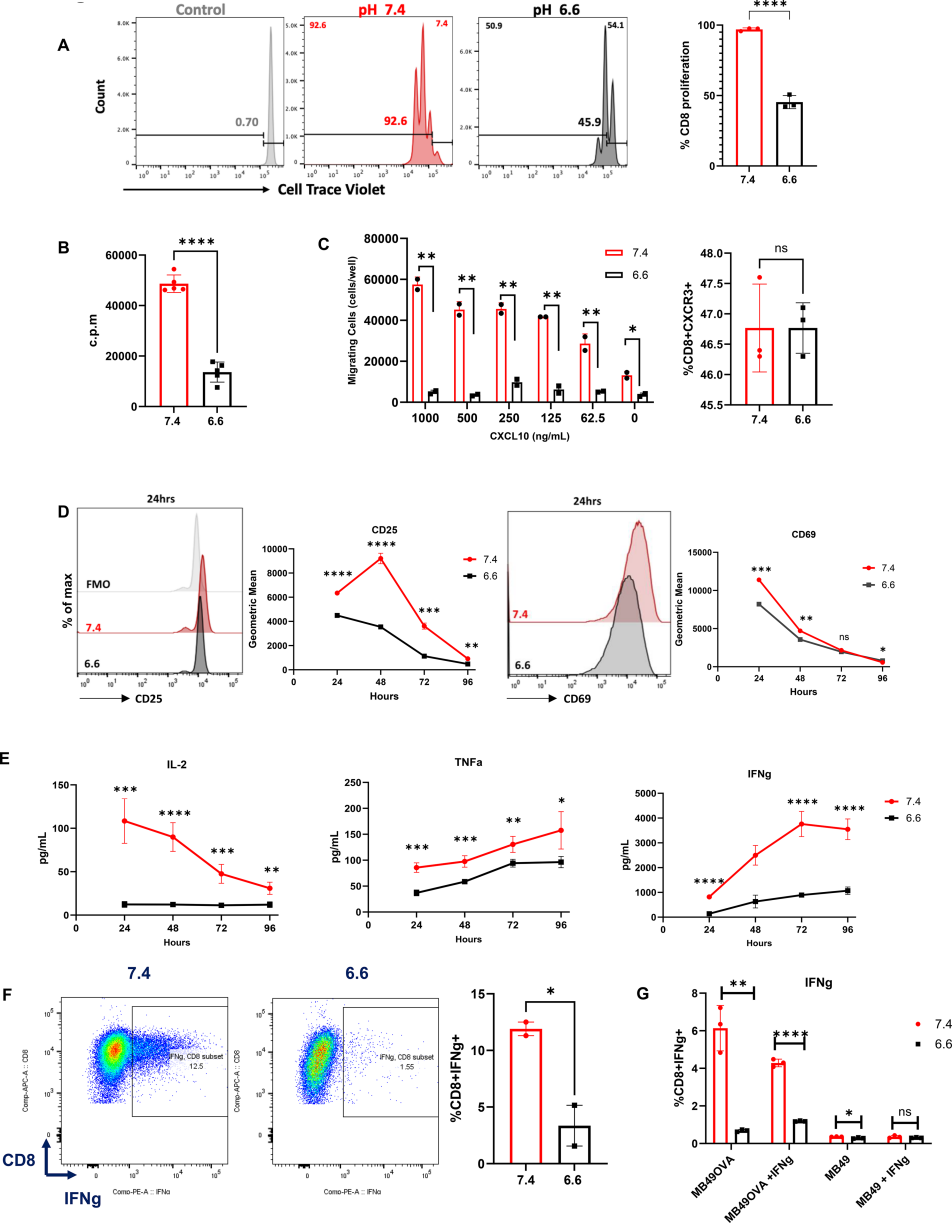
Acidity inhibits T-cell function. Assessment of T-cell proliferation. **(A)** Representative histograms and summary data showing CTV dilution of stimulated OT-I T cells at 72 h in pH 7.4 vs. 6.6 media. **(B)** Thymidine incorporation in pH 7.4 and 6.6 at 72 h post-stimulation. **(C)** Assessment of T-cell migration. Migration of OT-I T cells in pH 7.4 vs. 6.6 media according to CXCL10 serial dilution and flow cytometry staining of CXCR3 on CD8^+^ T cells 2 h post-incubation at pH 7.4 vs. 6.6. Two experiments were performed and each experiment contained two biological replicates for each CXCL10 concentration. **(D)** Representative histograms and summary graph of flow stained pmel T cells at 24, 48, 72 and 96 h post-stimulation for the activation markers CD25 and CD69 in pH 7.4 vs. 6.6 media. **(E)** Supernatant from stimulated pmel T cells analyzed via LEGENDplex for secreted IL-2, TNF-α, and IFN-γ levels after 24, 48, 72, and 96 h in pH 7.4 vs. 6.6 media. **(F)** Representative dot plot and summary graph of stimulated OT-I T cells in pH 7.4 vs. 6.6 media stained for intracellular IFN-γ (*p* < 0.0431). Assessment of T-cell reactivity via IFN-γ production. **(G)** Summary data showing the coculture of OT-I T cells at a 10:1 ratio with either MB49OVA, IFN-γ-pretreated MB49OVA, MB49, or IFN-γ-pretreated MB49 in pH 7.4 vs. 6.6 media. Results are one of two to four independent experiments, *n* = 2–5. Analysis by independent *t*-test (*p < 0.05; **p < 0.01; ***p < 0.001; ****p < 0.0001).

To investigate the effects of acidity on T cells, we first measured the proliferation of T cells stimulated in either pH 6.6 or pH 7.4 media. OT-I T cells were cultured with 5 μg/mL of OVA_SIINFEKL_ peptide for 72 h. Using CellTrace Violet (CTV) dilution, CTV-stained OT-I T cells stimulated at pH 6.6 showed significantly lower CTV dilution compared to pH 7.4, indicating reduced proliferative capacity (**Figure 1A**). To further confirm this, the thymidine proliferation assay showed that T cells cultured at pH 6.6 for 72 h had 3.5 times lower proliferation than T cells cultured at pH 7.4 (**Figure 1B**). Overall, T cells stimulated in acidity were less proliferative in comparison to those stimulated at physiological pH. We next assessed T-cell migration in acidity using Transwell assays with serial dilutions of CXCL10. At every CXCL10 dilution, migration was lower under acidic conditions vs. physiologic conditions (**Supplementary Figure 1A**; **Figure 1C**). We observed similar results with OT-II T cells, with unstimulated and stimulated CD4^+^ T cells cultured in pH 6.6 migrating less in response to CXCL10 compared to pH 7.4 (**Supplementary Figure 1B**). To investigate whether reduced CXCR3 (the receptor for CXCL10) expression caused these differences, OT-I T cells were cultured in different pH media for 2 h, and CXCR3 levels measured by flow cytometry remained unchanged (**Figure 1C**), indicating that chemokine receptor loss does not account for migration differences between acidic and physiological conditions.

We then examined the effect of acidity on T-cell activation. Pmel CD8^+^ T cells were stimulated with gp100 peptide and IL-2 at pH 6.6 or 7.4. Cells and supernatants were collected at various timepoints post-stimulation. Cells were stained for activation markers CD25 and CD69 and analyzed by flow cytometry. For CD25 expression, the geometric mean was much lower at pH 6.6 across all timepoints, with the largest difference at 48 h post-stimulation. CD69 expression was lower at pH 6.6 at 24 and 48 h compared to pH 7.4 (**Figure 1D**). Similar results were observed with OT-I CD8^+^ T cells stimulated with SIINFEKL peptide, where cells cultured at pH 6.6 showed lower activation than cells cultured at pH 7.4 at 24 and 48 h (**Supplementary Figures 1C, D**).

We next measured the effect of acidity on effector cytokine production. gp100-stimulated pmel CD8^+^ T cells were cultured at pH 6.6 or 7.4, and supernatants were collected at various timepoints. Cytokines were measured using the LEGENDplex Assay. Pmel T cells stimulated at pH 6.6 produced significantly lower IL-2, IFN-γ, and TNF-α across all timepoints (**Figure 1E**). Similarly, OT-I T cells were stimulated with OVA-SIINFEKL peptide for 24 h at pH 6.6 or 7.4 and treated with protein transport inhibitor 6 h before harvesting. The pH 6.6 condition yielded 3.5 times fewer CD8^+^IFN-γ^+^ T cells than pH 7.4. Also, less TNF-α and granzyme B were produced at pH 6.6 compared to 7.4 (**Figure 1F**; **Supplementary Figure 1E**), further supporting reduced T-cell effector functions at low pH.

Next, we evaluated the ability of T cells to recognize the OVA antigen expressed by MB49OVA bladder tumor cells. T cells isolated from OT-I spleens were cocultured with IFN-pretreated MB49OVA cells at pH 6.6 or 7.4 for 24 h. T cells were stained for intracellular IFN-γ. Fewer CD8^+^IFN-γ^+^ OT-I cells were measured at pH 6.6 than at pH 7.4. There was no reaction to parental MB49 cells at either pH (**Figure 1G**; **Supplementary Figure 1F**). These results support the suppressive role of acidosis on T cells in tumor microenvironmental conditions and are corroborated by our previous studies (17, 22). In addition, prior studies have shown that acidic extracellular pH restricts T-cell proliferation and suppresses the production of key effector cytokines, including IFN-γ, IL-2, and TNF-α, consistent with the inhibitory effects observed in our study (23, 24).

### RNA sequencing and pathway analysis

**Figure 2:**
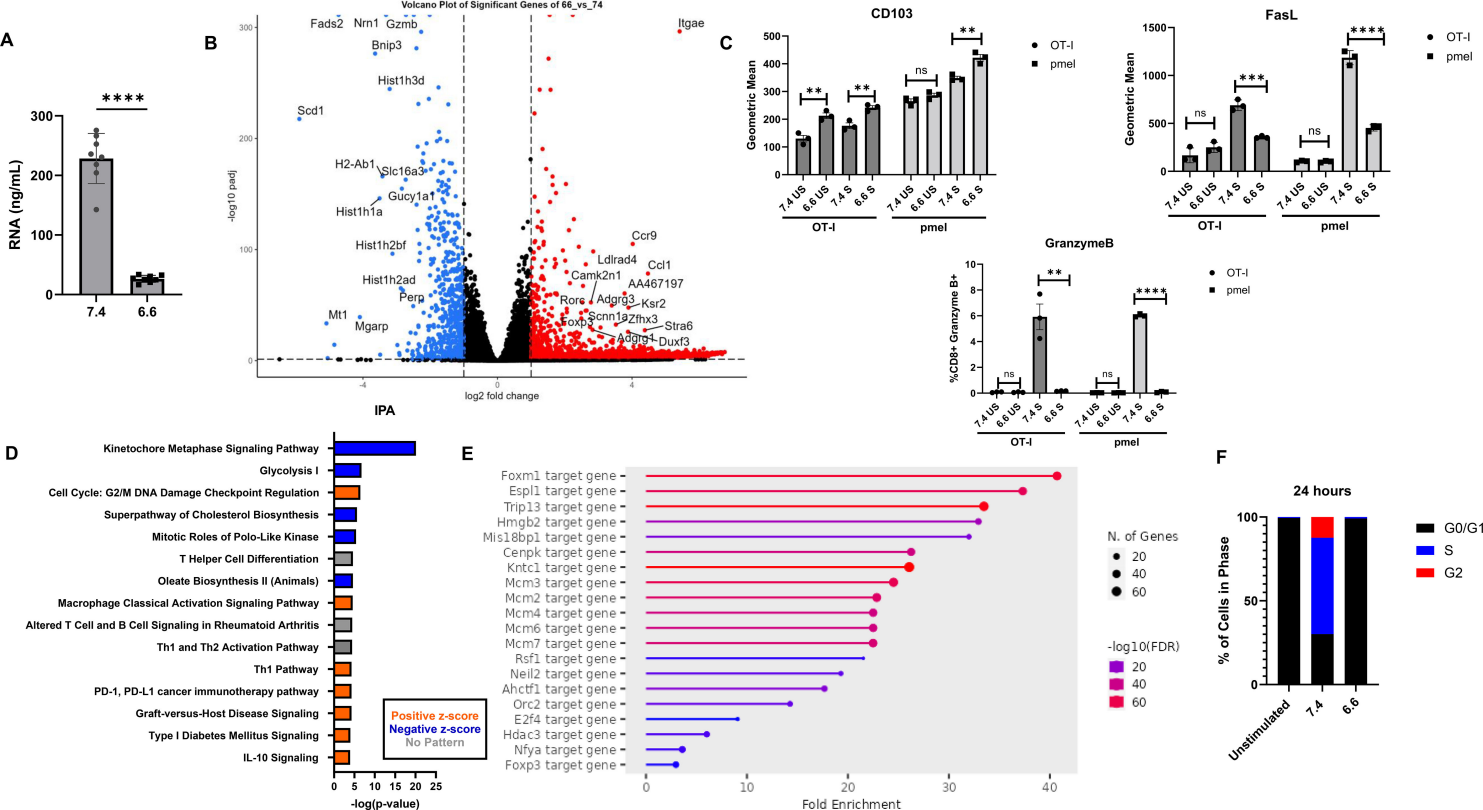
Differentially expressed genes in acidity. **(A)** Summary graph showing the concentration of RNA isolated from stimulated T cells at pH 7.4 vs 6.6. **(B)** Volcano plot showing protein coding DEGs, with genes in red showing increased expression in pH 6.6 in comparison to pH 7.4 and genes in blue have reduced expression in pH 6.6. Genes represented have padj<0.05 and log2fold<-1 or log2fold>1. Differentially expressed genes were validated by flow cytometry **(C)** Expression levels of CD103, FasL and Granzyme B on OT-I/ pmel CD8+ T cells stimulated at pH 7.4 vs 6.6. Analysis by independent t- test; (**p<0.01; ***p<0.001; ****p<0.0001). Pathway analysis of RNA sequencing dataset and metabolomics show **(D)** Top 15 pathways identified by Qiagen Integrated Pathway Analysis (IPA) with z-score direction. **(E)** Enriched target genes using ShinyGO 0.77 and TF.Target.RegNetwork pathway database. **(F)** Cell cycle analysis of stimulated pmel T cells at pH 7.4 and 6.6 represented as percentages were quantified using ModFit LT (Verity Software House). Unstimulated T cells at 24 hours were used as control. Experiment was repeated 2×.

Having shown that acidity affects T-cell functionality, we assessed transcriptional differences between T cells cultured at both pH conditions using RNA sequencing. CD8^+^ pmel T cells stimulated for 48 h yielded lower RNA concentrations at pH 6.6 than at pH 7.4 (**Figure 2A**). Even in unstimulated T cells, lower RNA concentrations were measured at pH 6.6 (**Supplementary Figure 2A**). The same number of viable cells was used to extract RNA for the analysis, and lower RNA levels at pH 6.6 were likely due to loss of effector function and suppressed transcriptional profile in response to reduced activation under acidic conditions. Protein-coding differentially expressed genes (DEGs) with padj <0.05 and log2fold >1 or ≤1 were significantly less prevalent at pH 6.6 compared to pH 7.4. The *Itgae*, *Vsir*, *Pmepa1*, *Skil*, and *Gpr68* genes were strongly expressed at pH 6.6, while *Gzmb*, *Fads2*, *Scd2*, *Pgk1*, and *Bnip3* were strongly expressed at pH 7.4 (**Figure 2B**; **Supplementary Figure 2B**). Because some genes are functionally relevant to T cells, we validated transcriptional differences at the protein level using flow cytometry. Using 48-h stimulated OT-I and pmel CD8^+^ T cells, we measured CD103 expression (encoded by *Itgae*). Geometric means were higher at pH 6.6 in both OT-I and pmel T cells compared to pH 7.4, including unstimulated conditions (**Figure 2C**). T cells stimulated at pH 6.6 produced significantly less granzyme B, while unstimulated conditions showed no differences (**Figure 2C**). Additionally, FasL levels were decreased at pH 6.6 compared to pH 7.4 upon stimulation, with no differences in unstimulated conditions (**Figure 2C**; **Supplementary Figure 2C**).

To identify enriched biological pathways, we applied pathway analysis to transcriptional data. We generated the top 15 relevant biological pathways using the Qiagen Integrated Pathway Analysis (**Figure 2D**). At pH 6.6, pathways were negatively enriched for glycolysis I, the super pathway of cholesterol biosynthesis, mitotic roles of polo-like kinase, and the kinetochore metaphase signaling pathway. In contrast, cell cycle: G2/M DNA damage checkpoint regulation was positively enriched at pH 6.6 (**Figure 2D**). These findings align with observed proliferation variations at pH 6.6, as most enriched pathways relate to cell division and the cell cycle.

Metabolomics data on pmel T cells activated for 48 h under corresponding pH levels identified 15 important pathways following RNA sequencing data integration, with purine metabolism, glycolysis or gluconeogenesis, and pyrimidine metabolism ranking the highest (**Supplementary Figure 2D**). This result aligns with RNA-seq pathway analysis, again highlighting disruptions to proliferative pathways.

### Cell cycle analysis

For T-cell metabolism and proliferation to meet energy and substrate needs, many proteins and enzymes must be rapidly produced (25). Given the impact of acidity on these processes, we believe DEGs associated with metabolism and proliferation may be displacing other relevant pathways. The metabolic alterations may also result functionally from decreased proliferation. To narrow the pathway scope, we investigated key transcriptional regulators. When comparing the pH 6.6 group’s negative significant DEGs to the TF.Target.RegNetwork murine database, Foxm1 emerged as the top target gene with 41-fold enrichment and enrichment FDR of 2.8E−68 (**Figure 2E**). Foxm1 is a primary cell cycle regulator promoting G1/S and G2/M progression. This transcription factor, overexpressed in many malignancies, controls the transcription of numerous cell cycling factors (26). Given decreased Foxm1 transcription at pH 6.6, we expected reduced transcription of significant cell cycling components. To determine whether cell cycle arrest occurs, we examined log2fold enrichment levels of key cell cycle regulators, including cyclins and CDKs (27). When cells are stimulated at pH 6.6, stratification of these components into corresponding cell cycle phases and comparison of their log2fold enrichment may indicate G1 phase arrest. Genes in red represent many cell cycle genes at pH 6.6 with log2fold enrichment ≤1 (**Supplementary Figure 2E**).

We performed cell cycle analysis on OT-I cells stimulated for 24 h to validate whether T cells stimulated at pH 6.6 undergo arrest at the G1 phase. T cells stimulated at pH 6.6 were arrested in the G0/G1 phase at 99.12%, very similar to unstimulated controls (99.48% in G0/G1). T cells stimulated at pH 7.4 showed 30.14% in G0/G1, 57.52% in the S phase, and 12.34% in the G2 phase (**Figure 2F**; **Supplementary Figure 2F**).

### Transdermal DYV800 bicarbonate therapy increases urine and intratumoral pH in MB49OVA tumors

**Figure 3:**
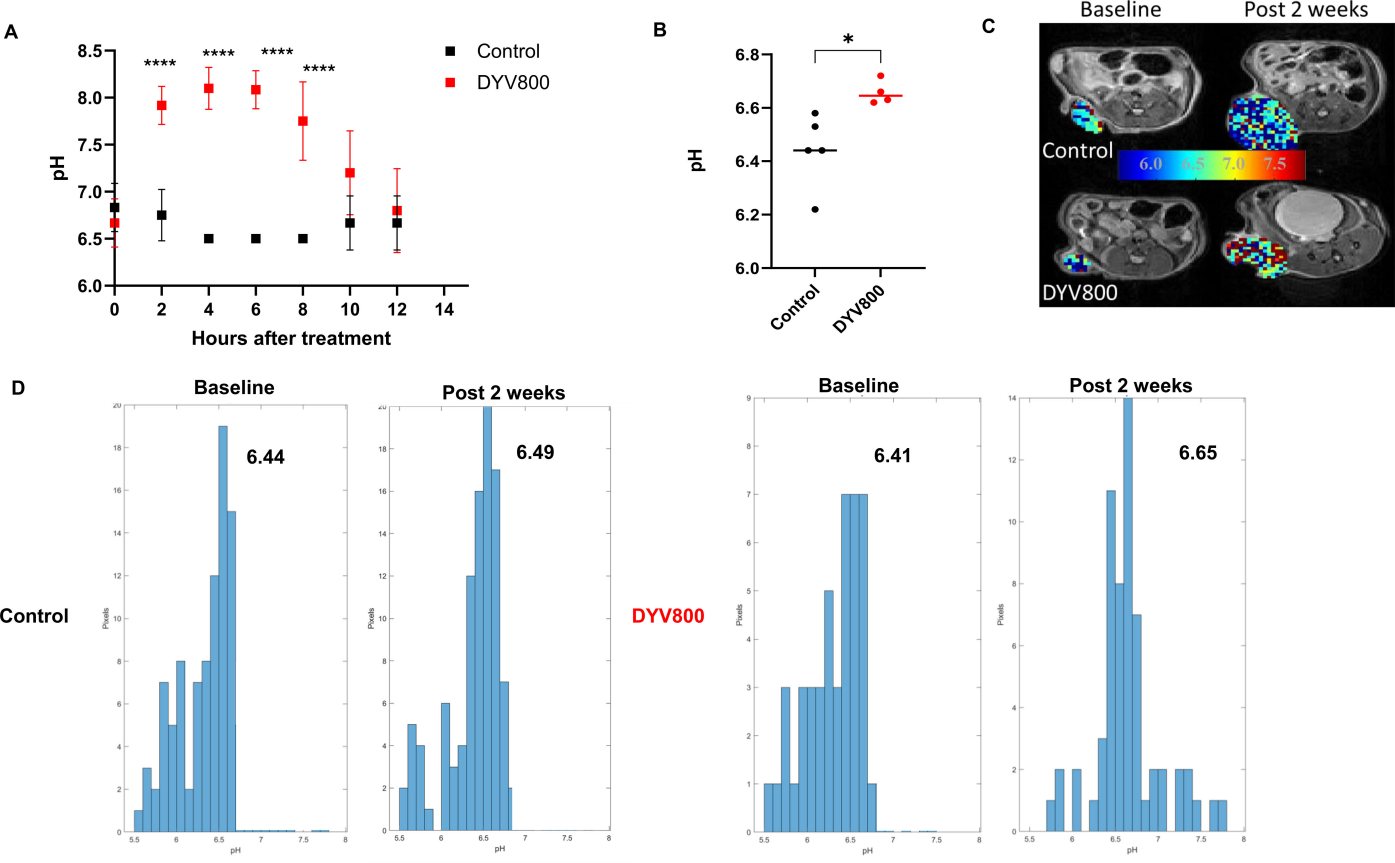
DYV800 elevates urine and tumor pH in subcutaneous MB49OVA bladder tumors. Mice were injected with MB49OVA cells SC. On day 7, mice were treated 1x with DYV800 cream on the tumor injection site. **(A)** Graph showing urine pH variations between control and treated mice over a 14-hour course after a one-time treatment at time 0; pH measurement taken using pH strips; *n*=6 per group. **(B)** Column bar graph showing increase in pHe values 4-6 hours after DYV800 treatment. Analysis by independent t- test (*p<0.05; ****p<0.0001; n=5). **(C)** Representative images for the pHe distribution across the tumor area (predominantly the center) with the 2D pHe-maps where color variations correspond to different pH values (please refer to the scalebar) allowing for the visualization of the spatial distribution of the pHe, and **(D)** with the relative histograms of the pH-maps showing the abundance (expressed as number of pixels) of the pHe clusters.

We investigated a novel treatment strategy to target acidity. DYV800 is a transdermal sodium bicarbonate cream delivery system. We first investigated the ability of DYV800 to alkalize urine after one treatment dose. Mice bearing SC MB49-OVA tumors were treated with 100 μL of DYV800 cream on the flank. Urine was collected at various timepoints. As expected, the urine of tumor-bearing mice was acidic at time 0, and one treatment alkalized urine for up to 10 h (**Figure 3A**). Furthermore, DYV800 is more efficient at alkalizing the urine of tumor-bearing mice compared to oral bicarbonate water (**Supplementary Figure 3A**). We used chemical exchange saturation transfer-magnetic resonance imaging (CEST-MRI) to measure tumor acidity at 4–6 h after a single application of cream. Mice bearing the MB49OVA tumor were treated with control or DYV800. DYV800 cream application significantly increased tumor pH (**Figure 3B**).

We further assessed tumor pHe changes after chronic DYV800 treatment. Mice bearing SC MB49OVA tumors were treated with DYV800 cream on the flank two times per day. Mice were imaged for baseline pH when tumors averaged 80–100 mm³, then at 2 weeks. Imaging sessions occurred 10–14 h after the last cream application. Representative images show pHe distribution across the tumor area (predominantly center) with 2D pHe maps displaying color variations corresponding to different pH values, visualizing spatial pHe distribution (**Figure 3C**) and relative histograms of pH maps showing abundance (expressed as pixel number) of pHe clusters (**Figure 3D**). Treated mice pHe values throughout the experiment (baseline, after 1 and 2 weeks of treatment) showed steady increasing trends (**Supplementary Figure 3B**). Tumor volumes extrapolated from MR images showed differences between the control and treated mice at baseline and after 2 weeks of treatment. Dimensions were homogeneous at baseline as expected, with a close to significant size reduction observed after 2 weeks of treatment (**Supplementary Figure 3C**).

### Transdermal DYV800 bicarbonate therapy reduces tumor burden

**Figure 4:**
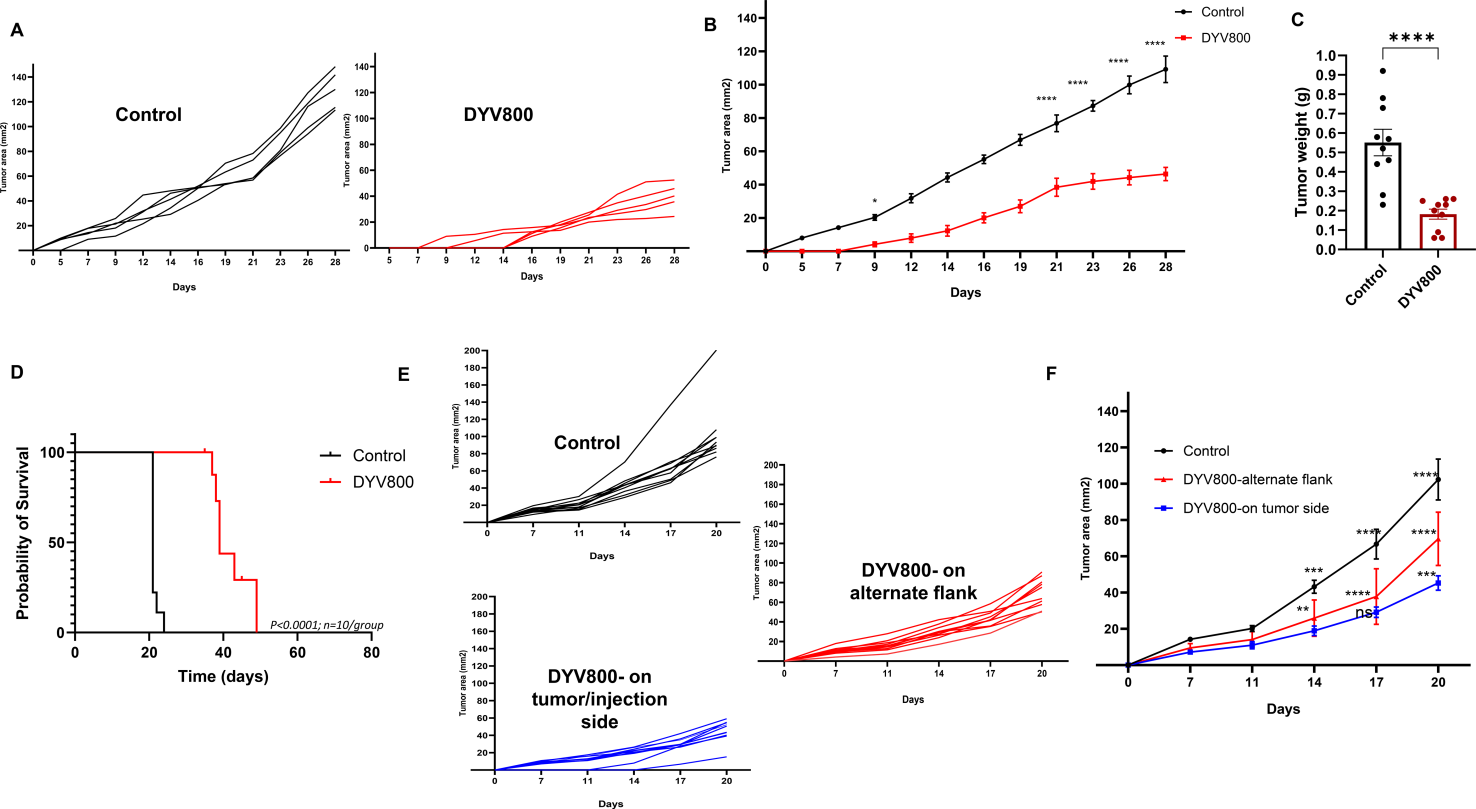
Transdermal DYV800 reduces tumor burden in subcutaneous MB49OVA tumors. C57BL/6 mice were treated 2x/day with 100ul of DYV800 24h post-MB49OVA tumor inoculation **(A–D)** or 7 days post-MB49OVA tumor inoculation **(E, F)**. **(A)** Individual tumor growth curves and **(B)** Summary tumor growth curve for Control and DYV800-treated groups. **(C)** Tumor weight of harvested tumors from Control and DYV800-treated groups (p<0.001). **(D)** Probability of survival in Control vs DYV800 group (p<0.001). **(E)** Tumors were established on the right flank, and DYV800 cream was applied either to the right flank (tumor side) or left flank (alternate flank). Individual growth curves from the three separate groups and **(F)** Summary growth curve of the three groups. Data is one of 2-3 independent experiments. Analysis by 2-way ANOVA (n=10; **p<0.01; ***p<0.001; ****p<0.0001).

Having shown that transdermal bicarbonate increases urine and intratumoral pH, we next investigated the antitumor effect of DYV800 on the growth of MB49OVA bladder tumors in C57BL/6 mice. Mice bearing MB49OVA tumors were treated with 100 μL of DYV800 applied to the flank near the tumor injection site 2×/day for 4 weeks, beginning 1 day post-inoculation. Tumors in DYV800 demonstrated a significantly reduced growth rate compared to the control, untreated mice (**Figures 4A, B**). DYV800-treated tumors showed reduced tumor weights (0.1820 ± 0.08 g) compared to controls (0.551 ± 0.22 g) (**Figure 4C**). This treatment also significantly increased the survival of tumor-bearing mice (**Figure 4D**).

To ensure that treatment efficacy was due to the bicarbonate in the cream, we treated mice with placebo (cream without buffer) or DYV800. A significant reduction in tumor progression and growth was observed with DYV800 compared to placebo (**Supplementary Figure 4A**), confirming that variations from repeated contact with the tumor site do not influence growth.

To evaluate the efficacy of DYV800 cream application in a model where tumors were more established, mice bearing MB49OVA tumors were treated with DYV800 starting on day 7 after tumor injection. DYV800 was applied 2× per day. When treatment started at 7 days post-tumor inoculation (after the tumor was established), the same effect on primary tumor growth was observed. On day 24, the tumor area of the DYV800-treated group (82.82 ± 43 mm^2^) was smaller compared to the control (133.8 ± 43.5 mm^2^) (**Supplementary Figure 4B**).

To investigate whether transdermal drug applications act locally or systemically, subcutaneous tumors were established on the right flank of C57BL/6 mice. On day 7 after tumor injection, mice were treated with 100 μL of DYV800 cream 2× per day either on the right flank (directly on the site of tumor injection) or on the alternate (left) flank. Regardless of the application site, tumor progression was delayed (**Figures 4E, F**), supporting the systemic effect of the DYV800 cream.

### DYV800 increases the activation and effector function of intratumoral CD8^+^T cells

**Figure 5:**
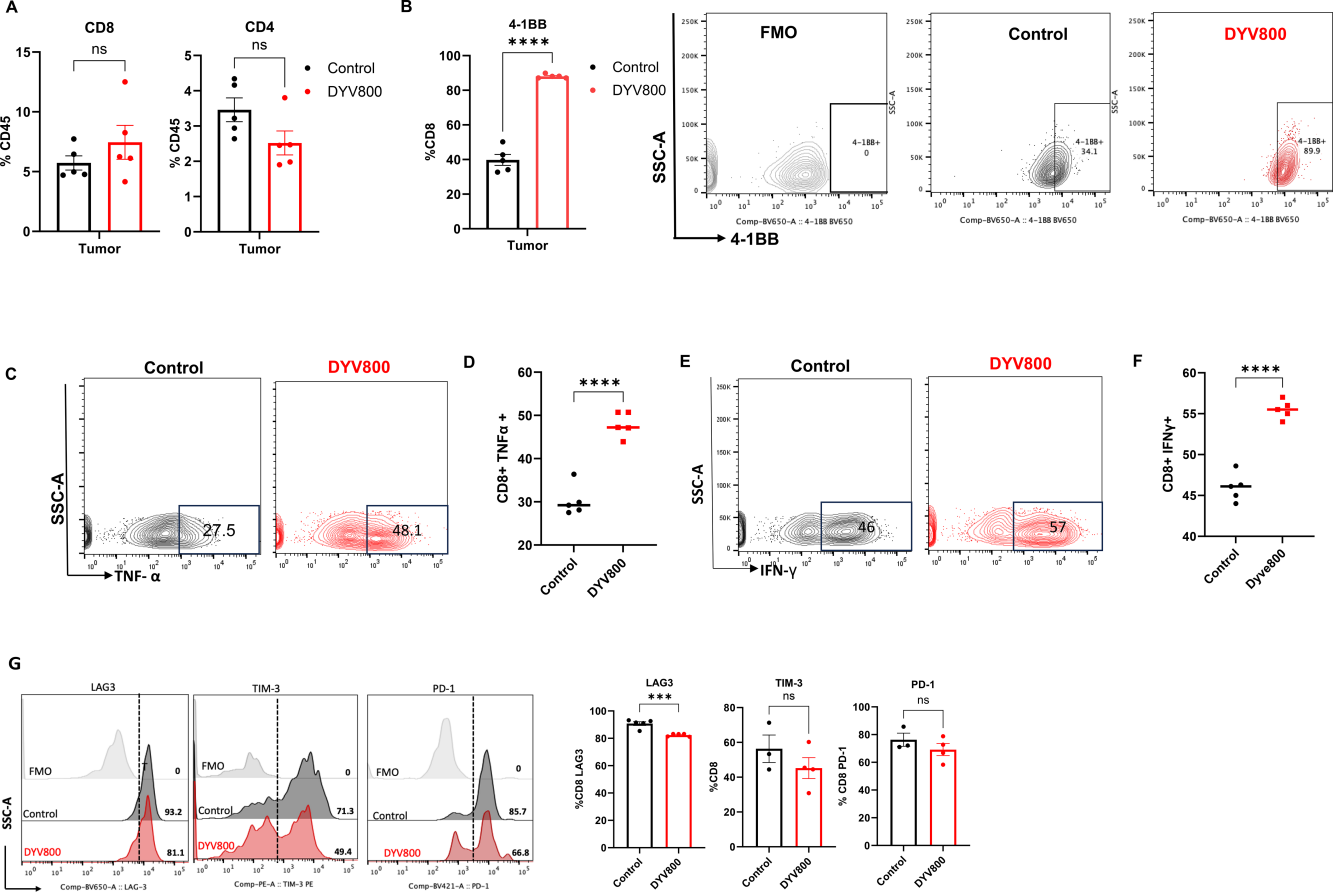
Transdermal DYV800 increases the activation and effector function of T cells. Tumor digest was analyzed via flow cytometry staining **(A)** Percentage of CD8^+^ and CD4^+^ T cells in the tumor expressed as a percentage of total CD45 Immune cell population **(B)** Dot plot and Summary graph showing 4-1BB expression on CD8^+^ T cells in the control and DYV800-treated groups. T cells were isolated from tumor cells, stimulated with cell stimulation cocktail plus protein transport inhibitor and stained for intracellular TNF-α and IFN-γ. **(C)** Dot plot and **(D)** Summary graph showing TNF-α levels in Control and DYV800 groups. **(E)** Dot plot and **(F)** Summary graph showing IFN-γ levels in Control and DYV800 groups. G. Representative histogram and summary data of PD-1, LAG-3 and TIM-3 on CD8+ T cells in the tumor. Data is one of 2-3 independent experiments. Independent t-test, two-tailed (*n*=5; ***p<0.001; ****p<0.0001).

To determine the effects of DYV800 therapy on intratumoral T cells, we established MB49OVA tumors in C57BL/6 mice and treated them with no cream (control) or DYV800 2× per day. T cells were analyzed in spleens and tumors via flow cytometry. Proportions of splenic and intratumoral CD8^+^ and CD4^+^ T cells were unchanged after 3 weeks of DYV800 treatment (**Figure 5A**; **Supplementary Figure 5A**), suggesting transdermal therapy has no direct effect on T-cell proportion in either the tumor or periphery. We assessed intratumoral T-cell activation status in untreated mice and mice treated with DYV800. The co-stimulatory molecule 4-1BB was expressed on CD8^+^ T cells in the tumor (**Figure 5B**). This trend was also observed on CD4^+^ T cells (**Supplementary Figure 5B**). We measured OX-40 expression, another T-cell activation marker, and observed no changes (data not shown).

To determine the effector function of intratumoral T cells, we measured inflammatory cytokine production after transdermal treatment. T cells isolated from tumors were stimulated with cell activation cocktail plus brefeldin A for 6 h, stained for intracellular cytokines, and assessed via flow cytometry. Stimulated CD8^+^ T cells in the DYV800 group demonstrated enhanced ability to produce IFN-γ and TNF-α (**Figures 5C–F**). This was confirmed in the supernatants after stimulating intratumoral T cells from each group for 24 h, showing increased IFN-γ and TNF-α levels in the treated groups (**Supplementary Figure 5C**).

Checkpoint molecules are quickly upregulated on T cells following activation, and their persistent surface expression can lead to effector T-cell dysfunction. Because of this, we measured the expression of checkpoint molecules on tumor CD8^+^ T cells and observed no major changes in PD-1, CTLA-4, and TIM-3 after 4 weeks of DYV800 treatment. The major difference was significantly reduced LAG3 on intratumoral CD8^+^ T cells (**Figure 5G**). Within the CD4^+^ T-cell compartment, PD-1 and LAG3 expression were significantly reduced (**Supplementary Figure 5D**). To evaluate whether T cells play a role in the antitumor effects of the DYV800 cream, bladder tumors were established in NSG mice. Mice were treated with DYV800 cream starting on day 7. No differences in tumor growth were observed in the control and treated groups, suggesting that the beneficial effects of DYV800 treatment may be mediated by T cells (**Supplementary Figure 5E**).

### Intratumoral CD8^+^ T cells demonstrate antigen specificity after DYV800 treatment

**Figure 6:**
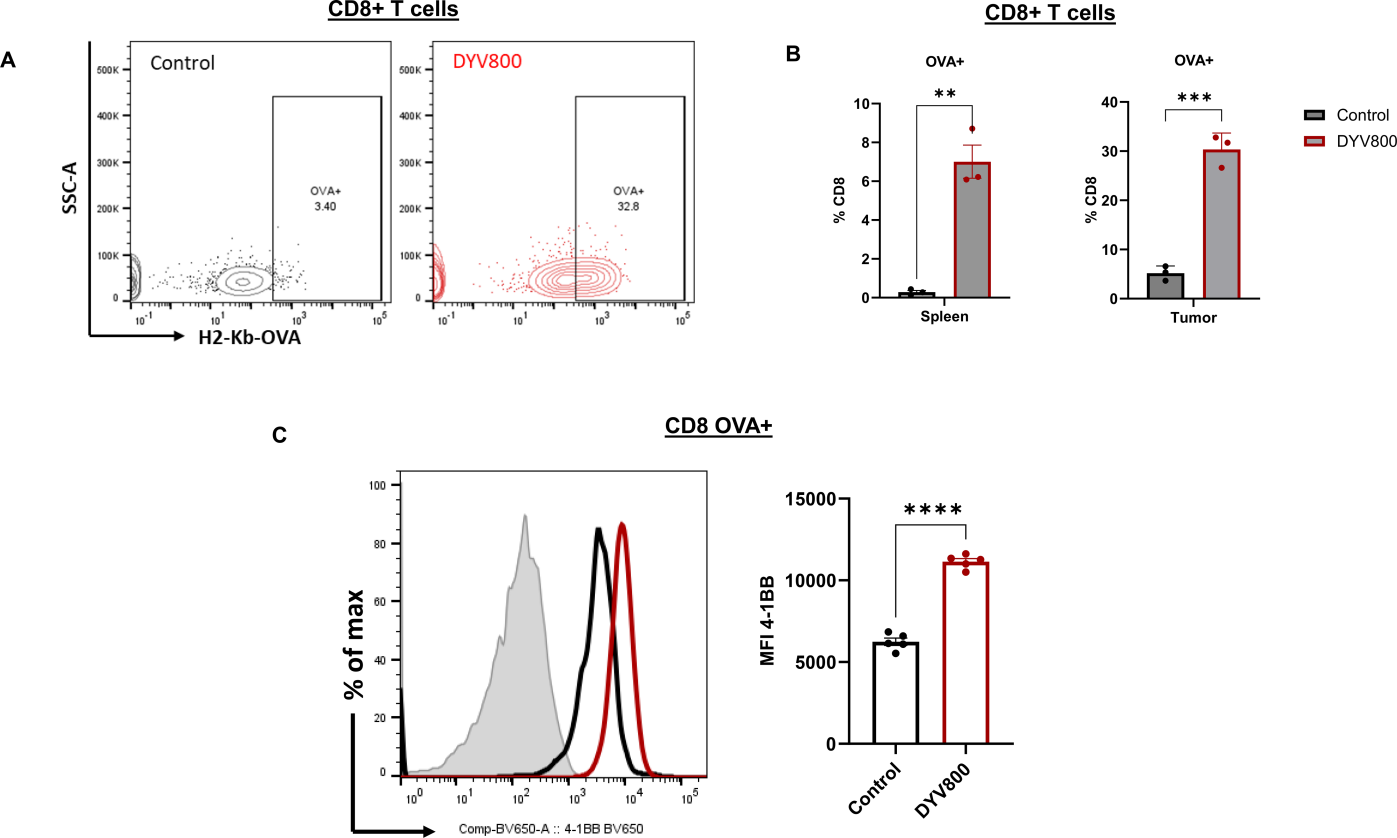
Transdermal DYV800 increases the percentage of OVA-specific CD8^+^T cells in tumor. **(A)** Representative dot plots of CD8^+^H2-Kb-OVAtetramer^+^ T cells in the tumor and **(B)** summary of CD8^+^OVAtetramer^+^ T cells in the spleen and tumor. **(C)** Representative histogram and summary of 4-1BB on OVA-specific CD8^+^ T cells in the tumor. Data is one of 2 independent experiments. Analysis by two-tailed independent t-test (n=5; **p<0.01; ***p<0.001; ****p<0.0001).

To further understand the effects of DYV800 on T-cell function, we assessed OVA-reactive CD8^+^ T cells in tumors via H2-Kb-OVA tetramer (OVA-tet^+^) staining (**Figures 6A, B**). Increased frequency of OVA-specific CD8^+^ T cells was measured in spleens and tumors of DYV800 cream-treated mice (**Figures 6A, B**). Comparable frequency of OVA-tetramer^+^ CD8^+^ T cells was measured in mice treated with oral bicarbonate or with DYV800 cream (**Supplementary Figure 6A**). OVA-tet^+^ CD8^+^ T cells in the DYV800-treated group also demonstrated significantly higher 4-1BB expression compared to controls, confirming activation of OVA-specific CD8^+^ T cells within tumors (**Figure 6C**). In addition, we observed a positive correlation between OVA-tet^+^ CD8^+^ T cells and tumor pH (*r*^2^ = 0.7368; *p* = 0.0031) (**Supplementary Figure 6B**). Taken together, these results support a role for DYV800 in enhancing antigen-specific CD8^+^ T-cell activation in bladder tumors, potentially in association with tumor pH modulation.

### Transdermal DYV800 slows tumor progression in orthotopic MB49OVA tumors

**Figure 7:**
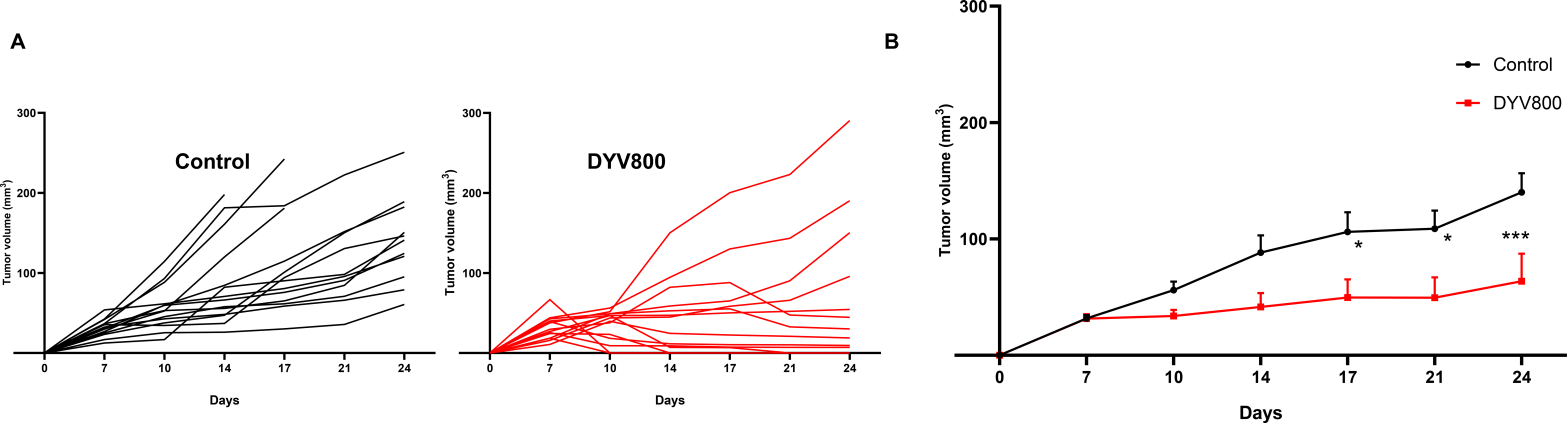
DYV800 slows tumor progression in an orthotopic model of bladder cancer: Tumor growth was monitored via ultrasound in already established orthotopic MB49OVA bladder tumors. Tumor growth curve of **(A)** control/untreated mice and DYV800-treated mice over the course of 24 days. **(B)** Combined growth curves for the control and DYV800-treated mice. Analysis by two-way ANOVA (*n* = 10; *p < 0.05; ***p < 0.001).

Having established that DYV800 drives an antitumor immune response in subcutaneous tumors, we assessed whether DYV800 affects tumor growth and/or pHe in orthotopic bladder tumors. Mice were implanted with orthotopic tumors via intravesical delivery of MB49OVA cells and monitored via ultrasound. After ultrasound confirmation of tumor establishment, DYV800 treatment was administered on the abdomen. Average bladder tumor volumes in the DYV800 group were smaller than those of untreated controls (**Figures 7A, B**). We also measured tumor pH via CEST-MRI in a single mouse per group. In this limited analysis, the pH of a DYV800-treated tumor was observed to be greater compared to an untreated or oral bicarbonate-treated mouse (**Supplementary Figure 7A**).

## Discussion

In our study, we assessed the effects of low pH on T cells, demonstrating impaired T-cell functionality under acidic conditions (pH 6.6) vs. physiological conditions (pH 7.4). Stimulation of murine T cells at pH 6.6 led to reduced overall function by affecting proliferative capacity, cytokine production, activation marker expression, and chemotactic migration to CXCL10 compared to pH 7.4. The effects of acidic pH on T-cell effector functions are corroborated by our previous study (17, 22) and other studies, affecting T-cell viability, proliferation, and IFN-γ production in mouse T cells (23, 24, 28) and in human cytotoxic T lymphocytes (29, 30). Other studies similarly reported impaired T-cell chemotaxis caused by tumor-derived lactic acid (29). The impairment in T-cell function corroborates other studies in the literature showing that these properties, including cytolytic functions, are restored upon pH buffering (17, 30). RNA sequencing revealed functional differences between physiological and acidic conditions at the transcriptional level. Pathway analysis of RNA sequencing and metabolomic datasets identified proliferation and metabolism pathways, including cell cycling, as major targets affected under pH 6.6 conditions. Validation showed significant T-cell arrest in the G0/G1 phase at pH 6.6. Further investigation of these pathways may provide mechanistic insights into molecules driving acidosis in immune and tumor cells. Given these profound acidosis effects on T-cell function, therapeutic interventions neutralizing tumor acidity represent a logical approach.

Buffer neutralization directly targets acidosis (31), and a previous preclinical study shows that sodium bicarbonate is a promising means to alkalize the acidic TME (13). However, in early phase I/II clinical trials, oral bicarbonate therapy has led to gastrointestinal disturbances, poor taste, poor compliance, limb edema, and vomiting (18). Therefore, clinically translatable interventions with minimal side effects are needed. Transdermal administration addresses these concerns and issues related to large oral bicarbonate dosages for acidity neutralization. We therefore evaluated a transdermal bicarbonate formulation to circumvent these limitations. In the MB49-OVA bladder tumor model, transdermal DYV800 therapy increased urine and extracellular tumor pH, inducing antitumor immune responses by slowing primary tumor growth and improving T-cell function. Acidic urine pH was neutralized for up to 10 h after one dose of DYV800, and CEST-MRI demonstrated increased extracellular tumor pH after a single application or 2 weeks of treatment with corresponding tumor volume decrease. Similar pH changes occurred regardless of cream application sites. Previous studies report that oral buffer therapy reduces metastasis but minimally affects primary tumor growth (14), while other studies show that tumor progression halts when oral buffer therapy precedes tumor inoculation (32). In this study, transdermal buffer therapy initiated after tumor inoculation significantly delayed tumor growth, reduced tumor weights, and increased survival in mice with subcutaneous or orthotopic tumors. Transdermal DYV800 delivery eliminated dosage-related issues associated with oral bicarbonate, making this approach more clinically translatable.

Beyond tumor growth and pH effects, we investigated whether antitumor efficacy was mediated through improved T-cell function. Previous studies demonstrate that acidic pH negatively affects T-cell immunity, while alkalization reverses these effects (33–35). Although T-cell numbers remained unchanged in tumors after DYV800 therapy, CD8^+^ T cells showed improved OVA-antigen recognition along with enhanced activation and effector function of CD8^+^ T cells. DYV800 could potentially combine with immune checkpoint inhibitors, yielding superior outcomes compared to monotherapy. Oral bicarbonate combined with anti-PD-1 blockade has been shown to induce better antitumor responses than anti-PD-1 monotherapy (17). Enhanced responses were also shown when L-DOS47 (a urease immunoconjugate capable of alkalizing acidity) was combined with anti-PD-1 therapy (36). Therefore, while transdermal alkalization may improve T-cell responses in our study, its combination with other immunotherapies may improve outcomes.

Together, these findings have significant translational relevance for bladder cancer treatment and solid tumors sharing acidic tumor microenvironments. DYV800 achieved robust alkalinization of urinary and intratumoral pHe, enhanced antigen-specific T-cell responses, and reduced tumor burden in subcutaneous and orthotopic models. Unlike prior systemic buffering approaches, DYV800 overcomes key translational barriers historically limiting oral bicarbonate therapy’s impractical dosing requirements and clinical applicability. By enabling therapeutically effective bicarbonate delivery through non-invasive, patient-friendly transdermal delivery, DYV800 represents a mechanistically novel intervention. Importantly, this strategy is grounded in clinical validation: Dyve Biosciences previously demonstrated transdermal bicarbonate administration feasibility in humans via a successful phase II trial of DYV700 for acute gouty arthritis (NCT04130204). This means that clinically meaningful bicarbonate doses can be transdermally delivered and that systemic buffering effectively treats acid-related pathologies. Collectively, these preclinical results position DYV800 as a first-in-class, systemically active, transdermal pH-modulating agent with potential integration into bladder cancer treatment paradigms—as monotherapy or combined with immune checkpoint inhibitors—and potentially extended to broader solid tumor spectra. By directly mitigating metabolic constraints imposed by tumor acidosis on T-cell function, DYV800 offers a promising, differentiated strategy to augment immunotherapeutic efficacy in patient populations with substantial unmet clinical need.

## Data Availability

In addition to the **Supplementary Material**, the original contributions presented in the study are publicly available. This data can be found here: https://www.ncbi.nlm.nih.gov/geo/query/acc.cgi?acc=GSE322498.

## References

[1] Bogdanov A Bogdanov A Chubenko V Volkov N Moiseenko F Moiseyenko V . Tumor acidity: From hallmark of cancer to target of treatment. Front Oncol. (2022) 12:979154. doi: 10.3389/fonc.2022.979154, PMID: 36106097 PMC9467452

[2] Estrella V Chen T Lloyd M Wojtkowiak J Cornnell HH Ibrahim-Hashim A . Acidity generated by the tumor microenvironment drives local invasion. Cancer Res. (2013) 73:1524–35. doi: 10.1158/0008-5472.CAN-12-2796, PMID: 23288510 PMC3594450

[3] Liberti MV Locasale JW . The Warburg effect: how does it benefit cancer cells? Trends Biochem Sci. (2016) 41:211–8. doi: 10.1016/j.tibs.2015.12.001, PMID: 26778478 PMC4783224

[4] Gatenby RA Gillies RJ . Why do cancers have high aerobic glycolysis? Nat Rev Cancer. (2004) 4:891–9. doi: 10.1038/nrc1478, PMID: 15516961

[5] Warburg O . On the origin of cancer cells. Science. (1956) 123:309–14. doi: 10.1126/science.123.3191.309, PMID: 13298683

[6] Tafech A Stéphanou A . On the importance of acidity in cancer cells and therapy. Biology. (2024) 13:225. doi: 10.3390/biology13040225, PMID: 38666837 PMC11048434

[7] Webb BA Chimenti M Jacobson MP Barber DL . Dysregulated pH: a perfect storm for cancer progression. Nat Rev Cancer. (2011) 11:671–7. doi: 10.1038/nrc3110, PMID: 21833026

[8] Gillies RJ Raghunand N Karczmar GS Bhujwalla ZM . MRI of the tumor microenvironment. J Magn Reson Imaging JMRI. (2002) 16:430–50. doi: 10.1002/jmri.10181, PMID: 12353258

[9] Garg T McMullen CK Leo MC O’Keeffe-Rosetti MC Weinmann S Nielsen ME . Predicting risk of multiple levels of recurrence and progression after initial diagnosis of nonmuscle-invasive bladder cancer in a multisite, community-based cohort. Cancer. (2021) 127:520–7. doi: 10.1002/cncr.33300, PMID: 33146913

[10] Alguacil J Kogevinas M Silverman DT Malats N Real FX García-Closas M . Urinary pH, cigarette smoking and bladder cancer risk. Carcinogenesis. (2011) 32:843–7. doi: 10.1093/carcin/bgr048, PMID: 21402590 PMC3106435

[11] Briguori C Roscigno G . Low urine pH: a new marker for contrast-associated acute kidney injury? EuroIntervention. (2022) 18:527–8. doi: 10.4244/EIJ-E-22-00026, PMID: 36134684 PMC10241262

[12] He Y Xue X Terkeltaub R Dalbeth N Merriman TR Mount DB . Association of acidic urine pH with impaired renal function in primary gout patients: a Chinese population-based cross-sectional study. Arthritis Res Ther. (2022) 24:32. doi: 10.1186/s13075-022-02725-w, PMID: 35078513 PMC8787907

[13] Ibrahim-Hashim A Cornnell HH Abrahams D Lloyd M Bui M Gillies RJ . Systemic buffers inhibit carcinogenesis in TRAMP mice. J Urol. (2012) 188:624–31. doi: 10.1016/j.juro.2012.03.113, PMID: 22704445 PMC3694604

[14] Robey IF Baggett BK Kirkpatrick ND Roe DJ Dosescu J Sloane BF . Bicarbonate increases tumor pH and inhibits spontaneous metastases. Cancer Res. (2009) 69:2260–8. doi: 10.1158/0008-5472.CAN-07-5575, PMID: 19276390 PMC2834485

[15] Azzarito T Lugini L Spugnini EP Canese R Gugliotta A Fidanza S . Effect of modified alkaline supplementation on syngenic melanoma growth in CB57/BL mice. PloS One. (2016) 11:e0159763. doi: 10.1371/journal.pone.0159763, PMID: 27447181 PMC4957829

[16] Astigiano S Puglisi A Mastracci L Fais S Barbieri O . Systemic alkalinisation delays prostate cancer cell progression in TRAMP mice. J Enzyme Inhib Med Chem. (2017) 32:363–8. doi: 10.1080/14756366.2016.1252760, PMID: 28095711 PMC6009900

[17] Pilon-Thomas S Kodumudi KN El-Kenawi AE Russell S Weber AM Luddy K . Neutralization of tumor acidity improves antitumor responses to immunotherapy. Cancer Res. (2016) 76:1381–90. doi: 10.1158/0008-5472.CAN-15-1743, PMID: 26719539 PMC4829106

[18] Gillies RJ Ibrahim-Hashim A Ordway B Gatenby RA . Back to basic: Trials and tribulations of alkalizing agents in cancer. Front Oncol. (2022) 12:981718. doi: 10.3389/fonc.2022.981718, PMID: 36452492 PMC9702334

[19] Bazargan S Bunch B Ojwang’ AME Blauvelt J Landin A Ali J . Targeting myeloid-derived suppressor cells with gemcitabine to enhance efficacy of adoptive cell therapy in bladder cancer. Front Immunol. (2023) 14:1275375. doi: 10.3389/fimmu.2023.1275375, PMID: 37901214 PMC10602731

[20] Jardim-Perassi BV Irrera P Lau JYC Budzevich M Whelan CJ Abrahams D . Intraperitoneal delivery of iopamidol to assess extracellular pH of orthotopic pancreatic tumor model by CEST-MRI. Contrast Media Mol Imaging. (2023) 2023:1944970. doi: 10.1155/2023/1944970, PMID: 36704211 PMC9836819

[21] Wu Y Zhou IY Igarashi T Longo DL Aime S Sun PZ . A Generalized Ratiometric Chemical Exchange Saturation Transfer (CEST) MRI Approach for Mapping Renal pH using Iopamidol. Magn Reson Med. (2018) 79:1553–8. doi: 10.1002/mrm.26817, PMID: 28686805 PMC5756701

[22] Wu H Estrella V Beatty M T-cells produce acidic niches in lymph nodes to suppress their own effector functions. (2020) Nat Commun. 11:4113. doi: 10.1038/s41467-020-17756-7. PMID: 32807791 PMC7431837

[23] Vuillefroy de Silly R Pericou L Seijo B Crespo I Irving M . Acidity suppresses CD8 + T-cell function by perturbing IL-2, mTORC1, and c-Myc signaling. EMBO J. (2024) 43:4922–53. doi: 10.1038/s44318-024-00235-w, PMID: 39284912 PMC11535206

[24] Nakagawa Y Negishi Y Shimizu M Takahashi M Ichikawa M Takahashi H . Effects of extracellular pH and hypoxia on the function and development of antigen-specific cytotoxic T lymphocytes. Immunol Lett. (2015) 167:72–86. doi: 10.1016/j.imlet.2015.07.003, PMID: 26209187

[25] Marchingo JM Cantrell DA . Protein synthesis, degradation, and energy metabolism in T cell immunity. Cell Mol Immunol. (2022) 19:303–15. doi: 10.1038/s41423-021-00792-8, PMID: 34983947 PMC8891282

[26] Koo C-Y Muir KW Lam EW-F . FOXM1: From cancer initiation to progression and treatment. Biochim Biophys Acta. (2012) 1819:28–37. doi: 10.1016/j.bbagrm.2011.09.004, PMID: 21978825

[27] Otto T Sicinski P . Cell cycle proteins as promising targets in cancer therapy. Nat Rev Cancer. (2017) 17:93–115. doi: 10.1038/nrc.2016.138, PMID: 28127048 PMC5345933

[28] Brand A Singer K Koehl GE Kolitzus M Schoenhammer G Thiel A . LDHA-associated lactic acid production blunts tumor immunosurveillance by T and NK cells. Cell Metab. (2016) 24:657–71. doi: 10.1016/j.cmet.2016.08.011, PMID: 27641098

[29] Fischer K Hoffmann P Voelkl S Meidenbauer N Ammer J Edinger M . Inhibitory effect of tumor cell-derived lactic acid on human T cells. Blood. (2007) 109:3812–9. doi: 10.1182/blood-2006-07-035972, PMID: 17255361

[30] Calcinotto A Filipazzi P Grioni M Iero M De Milito A Ricupito A . Modulation of microenvironment acidity reverses anergy in human and murine tumor-infiltrating T lymphocytes. Cancer Res. (2012) 72:2746–56. doi: 10.1158/0008-5472.CAN-11-1272, PMID: 22593198

[31] Wada H Hamaguchi R Narui R Morikawa H . Meaning and significance of “Alkalization therapy for cancer. Front Oncol. (2022) 12:920843. doi: 10.3389/fonc.2022.920843, PMID: 35965526 PMC9364696

[32] Bailey KM Wojtkowiak JW Cornnell HH Ribeiro MC Balagurunathan Y Hashim AI . Mechanisms of buffer therapy resistance. Neoplasia. (2014) 16:354–364.e3. doi: 10.1016/j.neo.2014.04.005, PMID: 24862761 PMC4094835

[33] Boedtkjer E Pedersen SF . The acidic tumor microenvironment as a driver of cancer. Annu Rev Physiol. (2020) 82:103–26. doi: 10.1146/annurev-physiol-021119-034627, PMID: 31730395

[34] Hosonuma M Yoshimura K . Association between pH regulation of the tumor microenvironment and immunological state. Front Oncol. (2023) 13:1175563. doi: 10.3389/fonc.2023.1175563, PMID: 37492477 PMC10363976

[35] Wang JX Choi SYC Niu X Kang N Xue H Killam J . Lactic acid and an acidic tumor microenvironment suppress anticancer immunity. Int J Mol Sci. (2020) 21:8363. doi: 10.3390/ijms21218363, PMID: 33171818 PMC7664620

[36] Jardim-Perassi BV Irrera P Oluwatola OE Abrahams D Estrella VC Ordway B . L-DOS47 elevates pancreatic cancer tumor pH and enhances response to immunotherapy. Biomedicines. (2024) 12:461. doi: 10.3390/biomedicines12020461, PMID: 38398062 PMC10886509

