# Supplementary material for: Transdermal bicarbonate buffer therapy increases intratumoral pH and elicits antitumor responses in bladder cancer
Source: Front Immunol. 2026 Mar 13;17:1706250. doi: 10.3389/fimmu.2026.1706250 (PMC13021593; doi:10.3389/fimmu.2026.1706250)
Supplement: Supplementary file 1 [file Table1.docx]

**Supplementary Figure Legend**

**Supplemental Figure 1:** **Acidity inhibits T cell function** **A.** Number of cells plated for OT-I T cell transwell migration assay. **B**. Migration of OT-II T cells in pH 6.6 vs 7.4 media. **C-E**. Flow cytometry of stimulated OT-I T cells showing **C.** Mean Fluorescence Intensity **(**MFI) of CD25 activation and **D.** MFI of CD69 activation status at 24, 48, and 72 hours in pH 6.6 and 7.4. **E.** MFI of Granzyme B and TNF-α on stimulated OT-I T cells after 24 hours in pH 6.6 and 7.4 media. **F**. Representative dot plot showing coculture of OT-I T cells at 10 :1 ratio with either MB49OVA, IFN-g-pretreated MB49OVA, MB49 or IFN-g pretreated MB49 in pH 7.4 VS 6.6 media. Analysis by independent t- test; (*=p<0.05; **=p<0.01; ***=p<0.001; ***=p<0.0001).

**Supplemental Figure 2**: **Differentially expressed genes and Pathway analysis dataset**. **A**. Summary graph showing the concentration of RNA isolated from unstimulated T cells at pH 6.6 vs 7.4. **B**. Heatmap of protein coding DEGs in pH 7.4 vs 6.6 conditions. Genes represented have padj<0.05 and log2fold<-1 or log2fold>1. **C.** Representative flow staining of CD103 and FasL on pmel CD8+ T cells stimulated at pH 6.6 and 7.4. **D.** Relevant metabolic pathways using Metaboanalyst Integrated Analysis, merging RNA sequencing and metabolomic data. **E**. Differentially expressed cell cycle genes and respective log2fold change. All genes used for pathway analysis were padj < 0.05, base mean >100, and log2fold >1 or log2fold < -1 (or otherwise specified). Genes shown in red have log2fold enrichment < -1. All metabolites used for pathway analysis were p < 0.05 and log2 ratio > 0.58 or log2 ratio < -0.58. **F**. Cell cycle analysis of pmel T cells stimulated at pH 6.6 vs 7.4. Representative flow cytometry plots showing differences in cell cycle distribution.

**Supplemental Figure 3: DYV800 elevates urine and tumor pH in subcutaneous MB49OVA bladder tumors. A.** Urine pH of tumor-bearing mice after no treatment (Control) or treatment with bicarb water or DYV800 (n=5)**. B.** pHe values of tumors in treated mice at baseline, post 1-and-2 weeks of DYV800 treatments. Analysis by 2-way ANOVA **C.** Tumor volumes measured with MRI reporting the differences in tumor size between control and DYV800 treated mice at baseline and after 2 weeks of treatment (*=p<0.05; **=p<0.01; ***=p<0.001; ****=p<0.0001).

**Supplemental Figure 4: Transdermal DYV800 reduces tumor burden in subcutaneous Mb49OVA tumors**. **A.** Subcutaneous tumors were established in C57BL/6 mice and mice were randomized into placebo or DYV800 groups. Tumor growth curve shows individual tumor growth of mice from each group. Treatment started 3 days after tumor injection. **B**. Subcutaneous tumors were established in C57BL/6 mice and mice were randomized into control and DYV800 treated groups. Treatment started 7 days after tumor injection. Data is one of two to three independent experiments. Analysis by 2-way ANOVA (ns=not significant, **=p<0.01; ***=p<0.001).

**Supplemental Figure 5: Transdermal DYV800 increases the activation and effector function of T cells**. Flow cytometry data showing **A**. Percentage of CD8+ and CD4+ T cells in the spleen expressed as a percentage of total CD45 Immune cell population **B.** Summary graphs showing 4-1BB expression on CD8+ T cells (spleen) and CD4+ T cells (spleen and tumor) of control and DYV800-treated groups. Legend plex assay was performed using supernatants from stimulated Intra-tumoral T cells **C.** Concentration (in pg/ml) of TNF-α and IFN-γ in Control and DYV800 groups. Flow cytometry data showing **D**. PD-1 and LAG-3 expression on CD4+ T cells in the tumor. **E.** Bladder tumors were established in NSG mice. Tumor growth curve of Control and DYV-treated NSG tumors. Analysis by independent t- test; (*=p<0.05; **=p<0.01; ***=p<0.001; ****=p<0.0001)

**Supplemental Figure 6: Positive correlation between pH and CD8+ OVA tetramer+ T cells.**  **A.** Flow cytometry showing increased CD8+H2-Kb-OVA tetramer+ T cells in the tumor of treated groups (Bicarb in water or DYV800) compared to Control. **B.** Association between pH and CD8+ H2-Kb-OVAtetramer+ T cells. Correlation was assessed using Pearson’s correlation, with correlation coefficients and p value indicated (n=9; 5 control and 4 DYV800 treated).

**Supplemental Figure 7**: **DYV800 alters intratumoral pHe in an orthotopic model of bladder cancer.** MRI images of mice (control, bicarb water-treated, or DYV800 treated) with orthotopic MB49OVA tumors. Representative images for the pHe distribution across the tumor area with the 2D pHe-maps where color variations correspond to different pH values (please refer to the scalebar) allowing for the visualization of the spatial distribution of pHe in the tumor.
