# Supplementary material for: Transdermal bicarbonate buffer therapy increases intratumoral pH and elicits antitumor responses in bladder cancer
Source: Front Immunol. 2026 Mar 13;17:1706250. doi: 10.3389/fimmu.2026.1706250 (PMC13021593; doi:10.3389/fimmu.2026.1706250)

Supplemental Figure 1

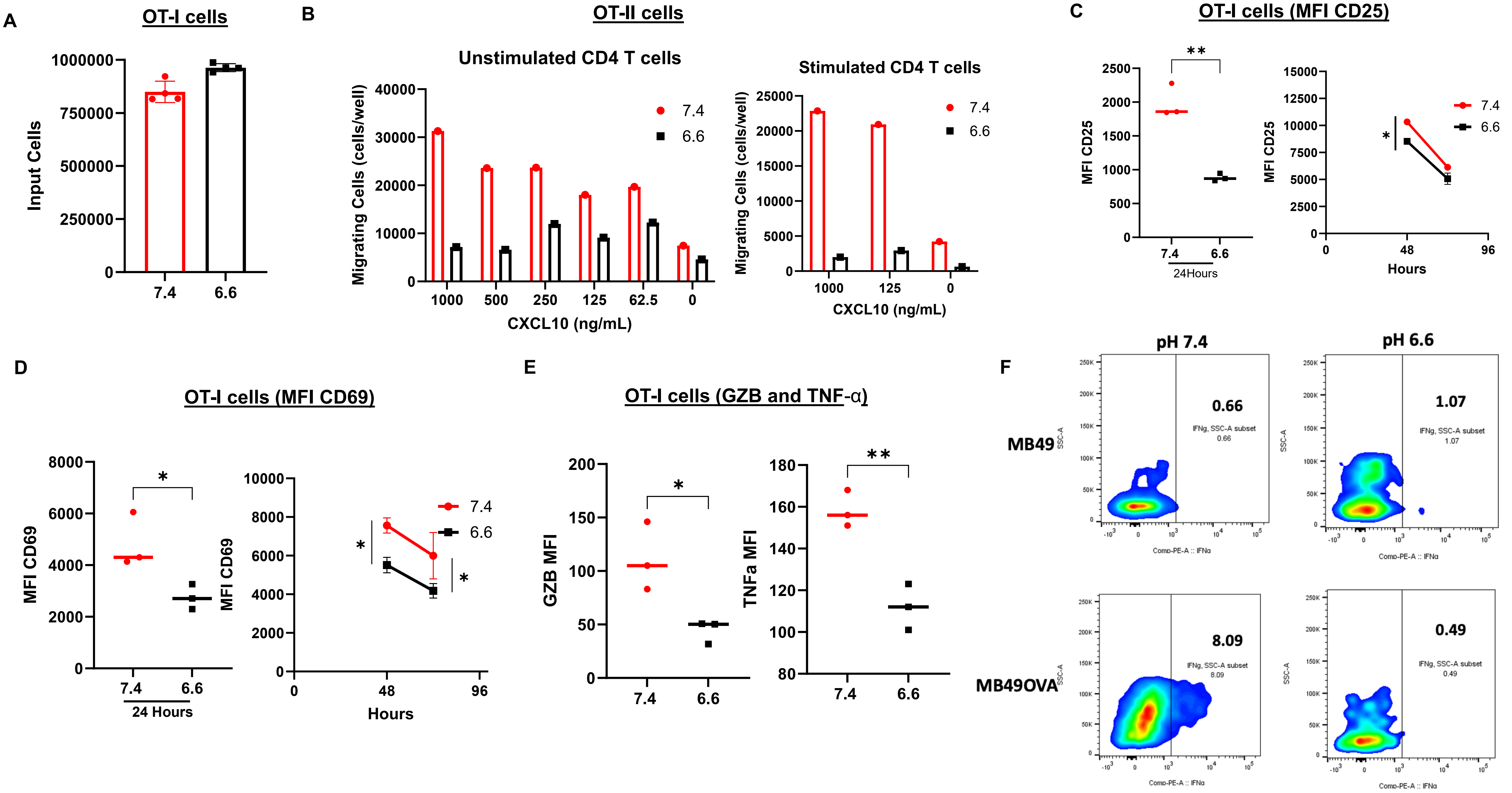

Supplemental Figure 2A-D

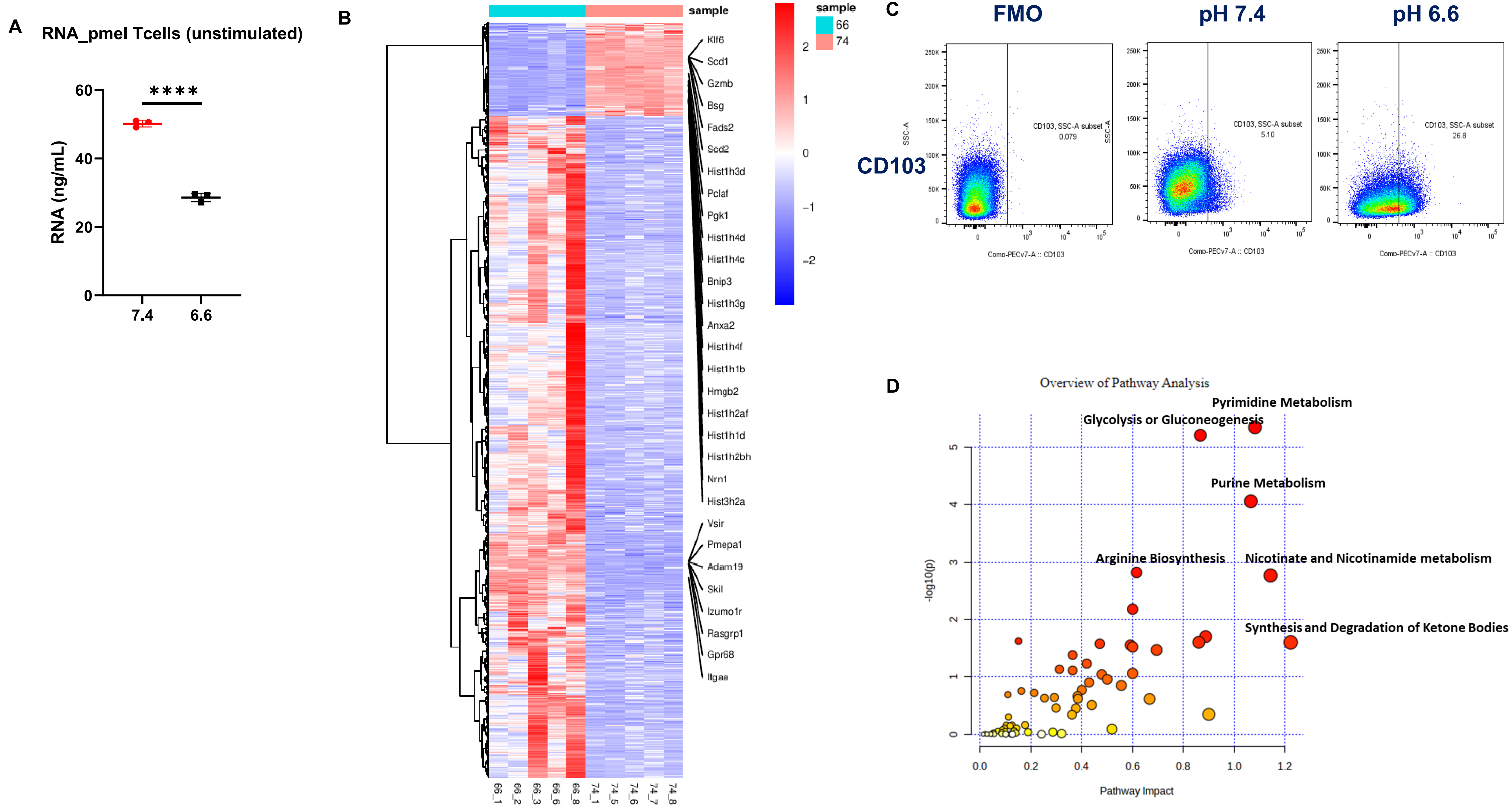

Supplemental Figure 2E-F

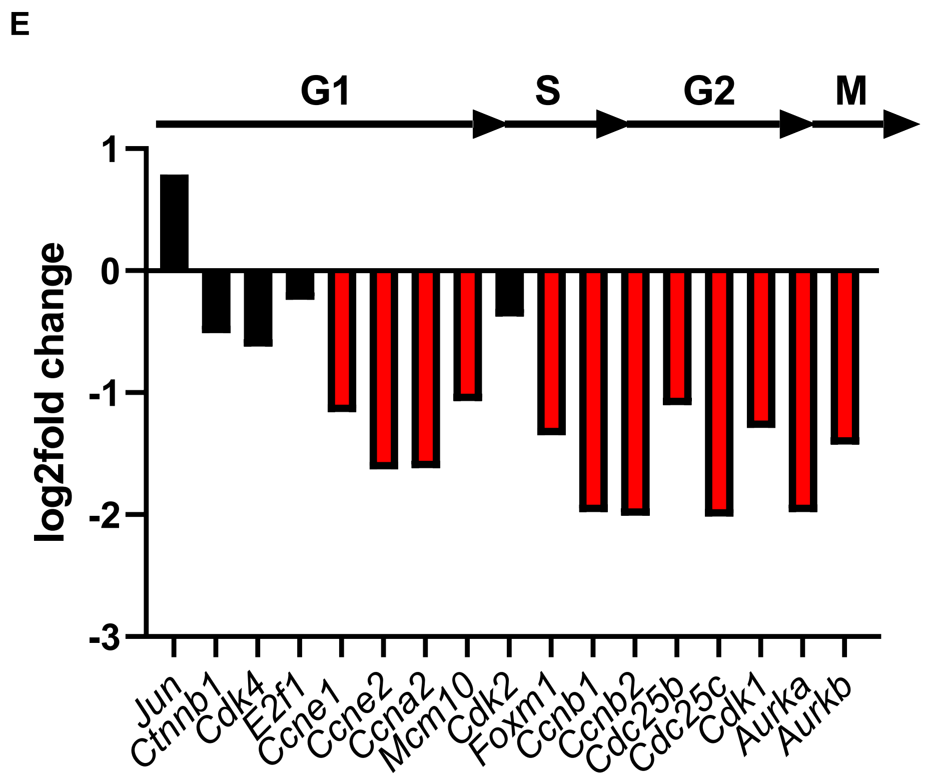

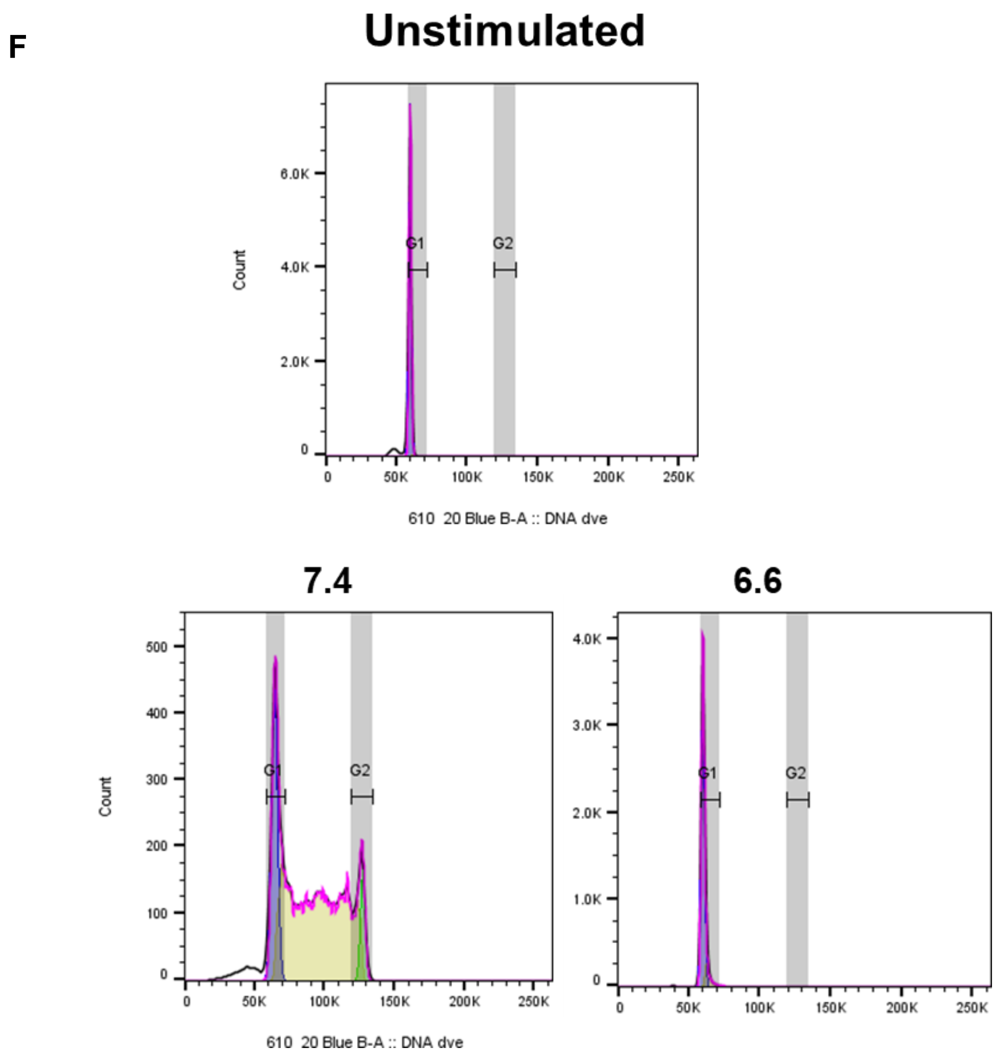

Supplemental Figure 3

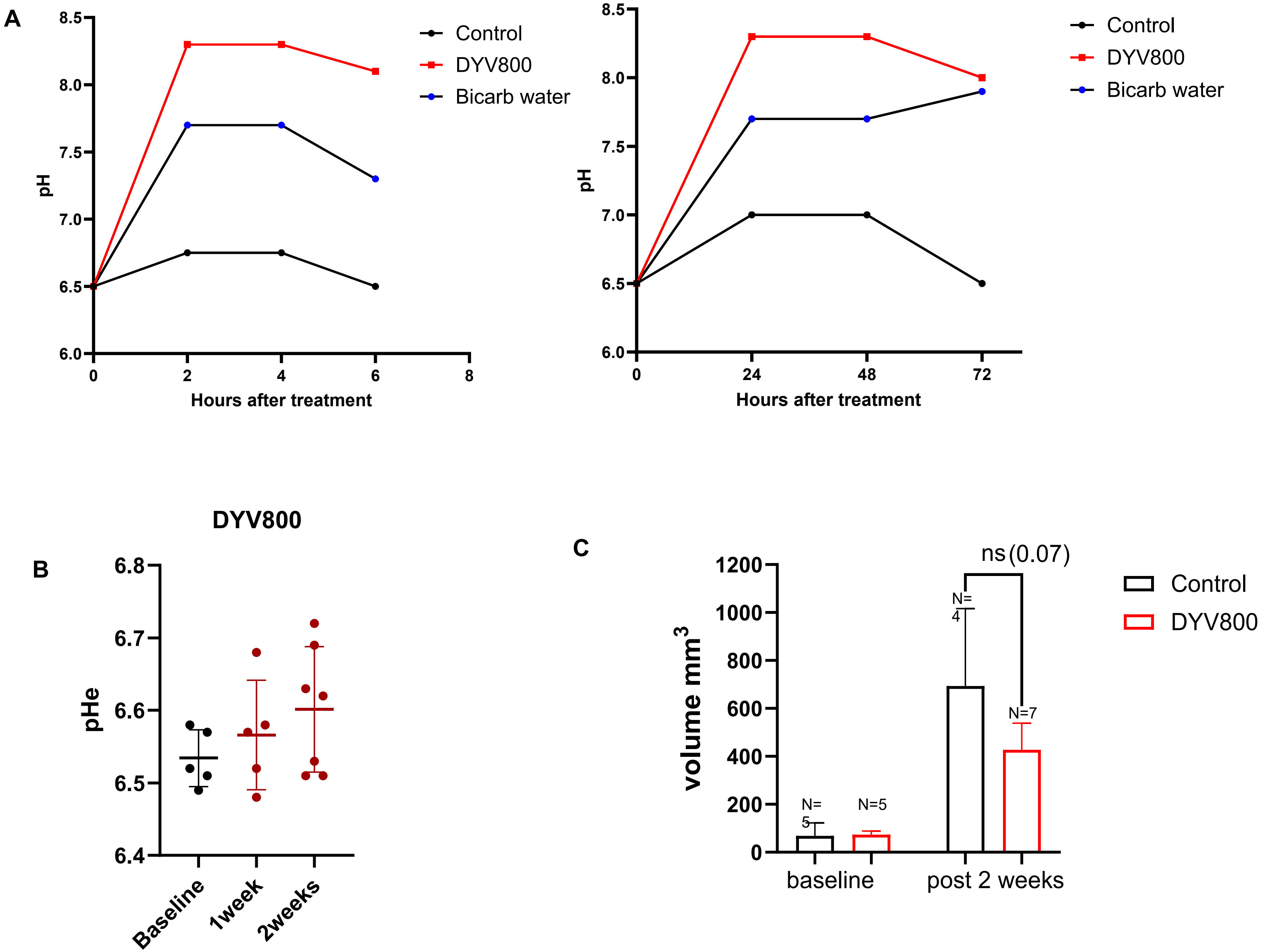

Supplemental Figure 4

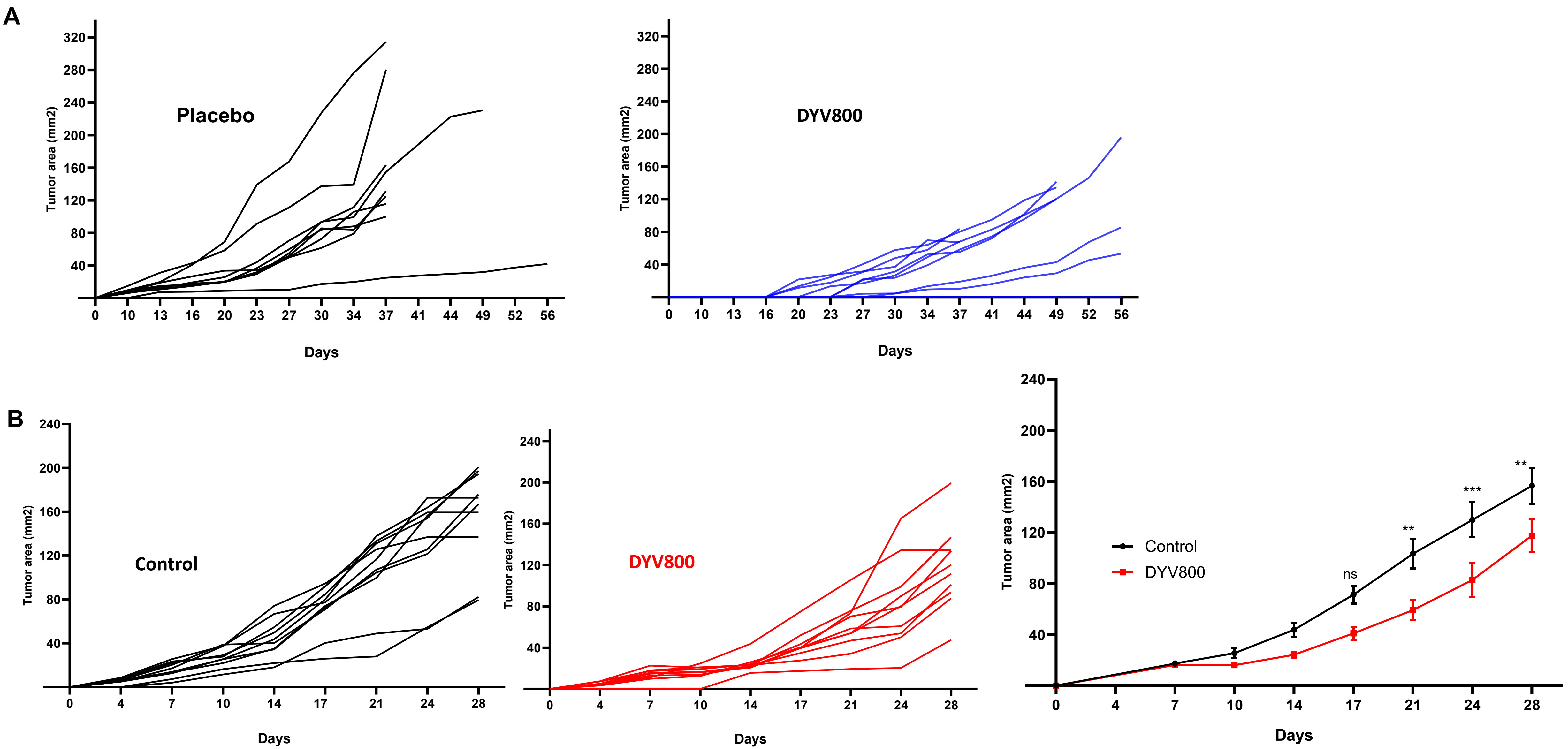

Supplemental Figure 5

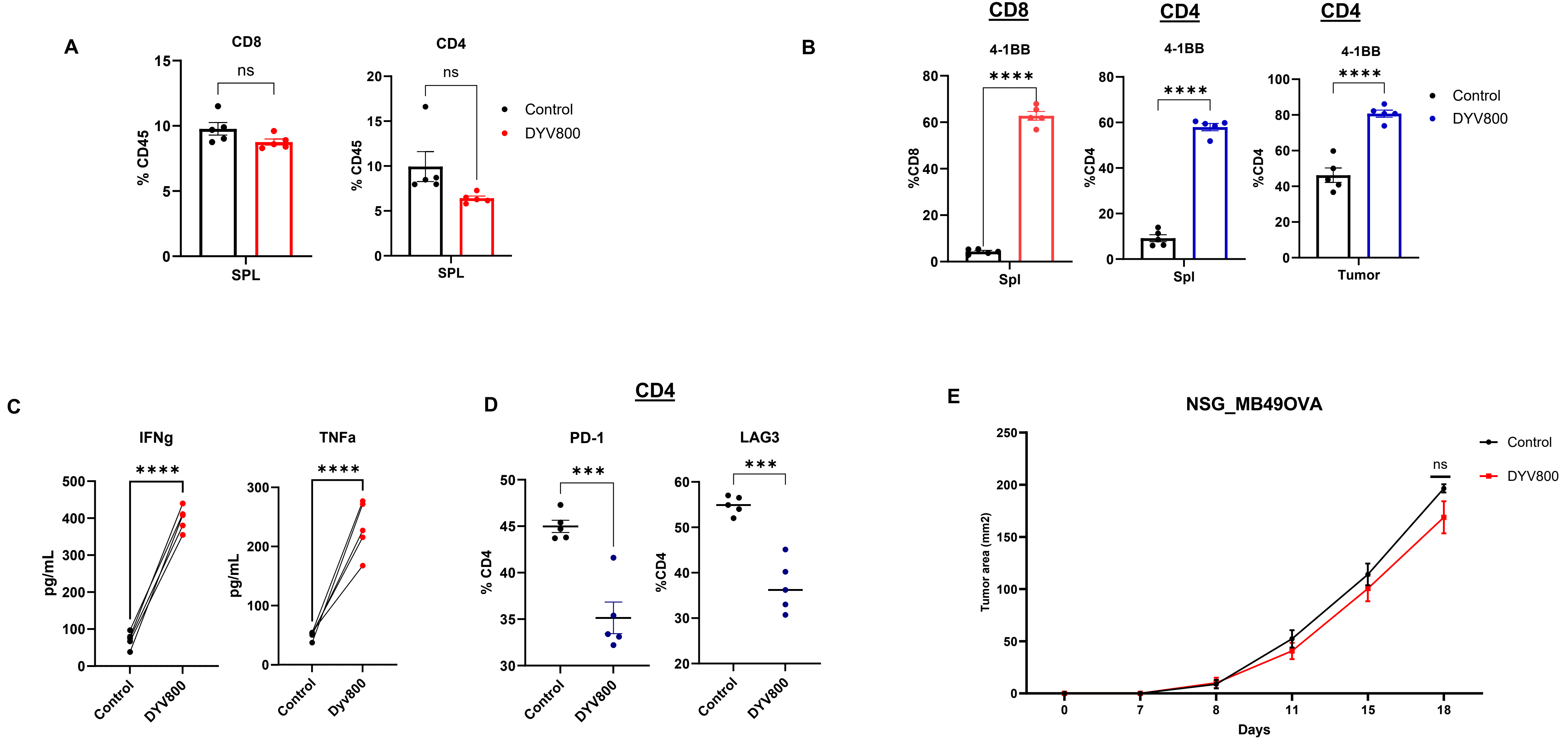

Supplemental Figure 6

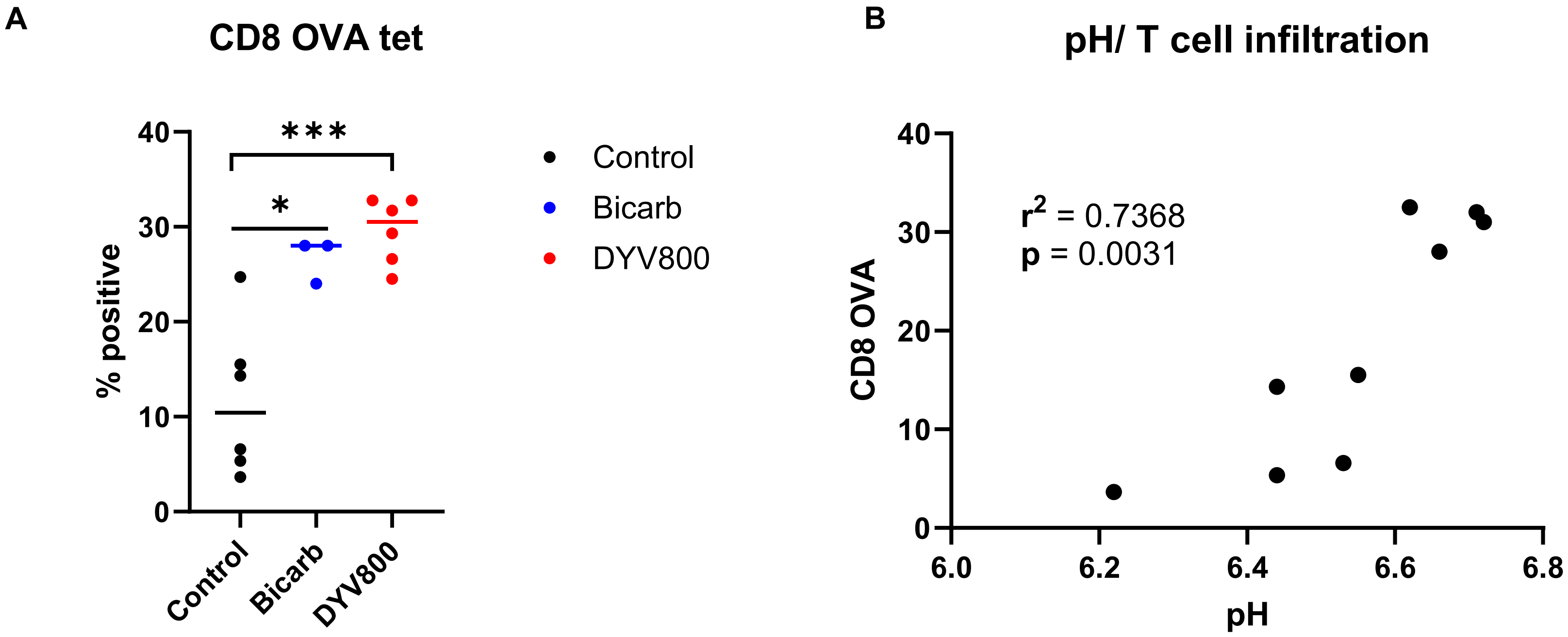

Pearson r	
r	0.8583
95% confidence interval	0.4517 to 0.9697
R squared	0.7368
P value	
P (two-tailed)	0.0031
P value summary	**
Significant? (alpha = 0.05)	Yes
Number of XY Pairs	9

Supplemental Figure 7

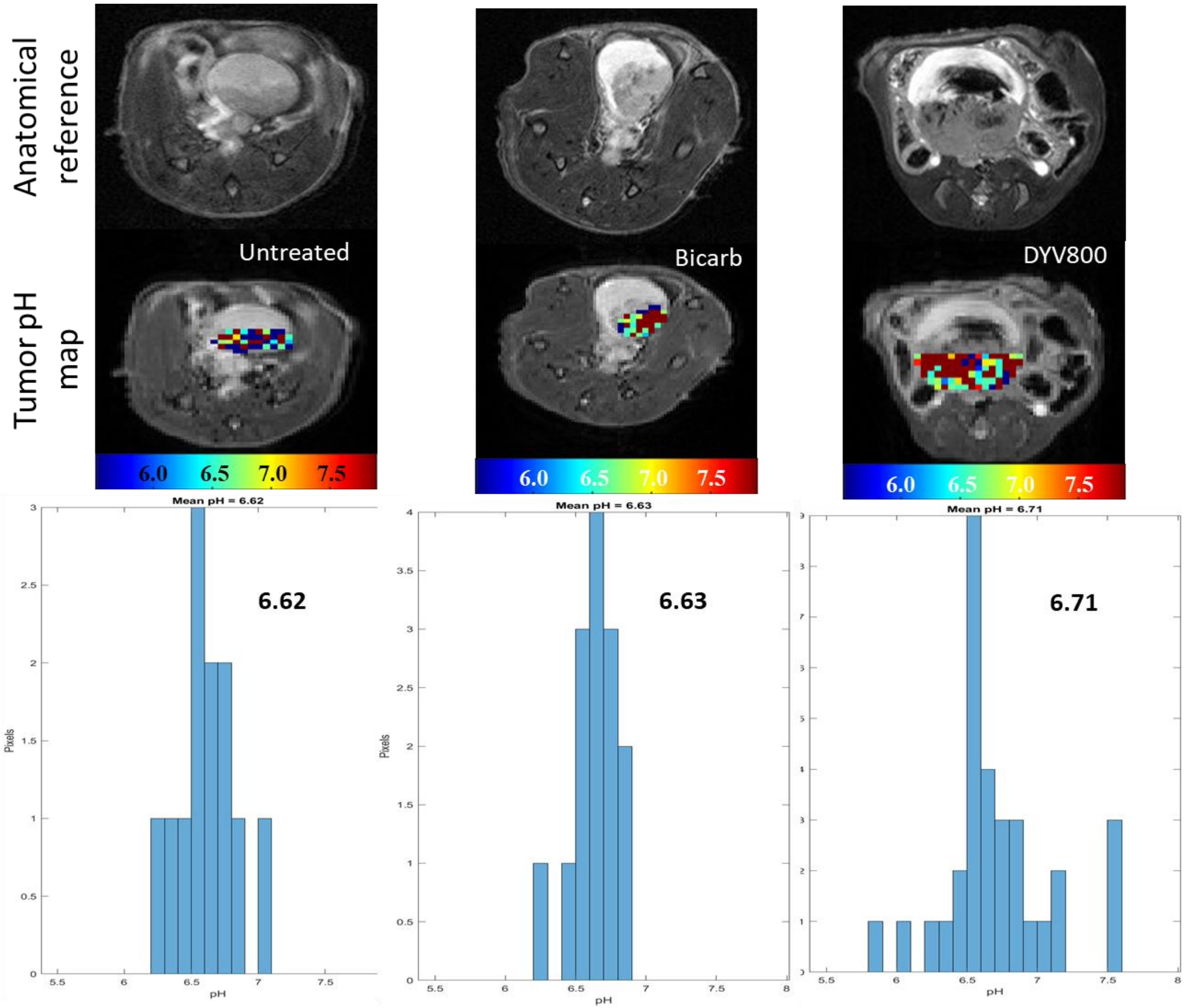

Supplement: Supplementary file 2 [file DataSheet1.pdf]
